# Genetic analysis of hsCRP in American Indians: The Strong Heart Family Study

**DOI:** 10.1371/journal.pone.0223574

**Published:** 2019-10-17

**Authors:** Lyle G. Best, Poojitha Balakrishnan, Shelley A. Cole, Karin Haack, Jonathan M. Kocarnik, Nathan Pankratz, Matthew Z. Anderson, Nora Franceschini, Barbara V. Howard, Elisa T. Lee, Kari E. North, Jason G. Umans, Joseph M. Yracheta, Ana Navas-Acien, V. Saroja Voruganti

**Affiliations:** 1 Missouri Breaks Industries Research Inc. Eagle Butte, SD, United States of America; 2 School of Medicine, University of Alabama at Birmingham, Birmingham, AL, United States of America; 3 Texas Biomedical Research Institute, San Antonio, TX, United States of America; 4 Public Health Sciences, Fred Hutchinson Cancer Research Center, Seattle, WA, United States of America; 5 Department of Laboratory Medicine and Pathology, University of Minnesota, Minneapolis, MN, United States of America; 6 Department of Microbiology, Ohio State University, Columbus, OH, United States of America; 7 Department of Microbial Infection and Immunity, The Ohio State University, Columbus, OH, United States of America; 8 Department of Epidemiology, University of North Carolina, Chapel Hill, NC, United States of America; 9 MedStar Health Research Institute, Hyattsville, MD, United States of America; 10 Georgetown-Howard Universities Center for Clinical and Translational Science, Washington, DC, United States of America; 11 College of Public Health, University of Oklahoma Health Sciences Center, Oklahoma City, OK, United States of America; 12 University of North Carolina, Gillings School of Global Public Health, Chapel Hill, NC, United States of America; 13 Department of Environmental Health Sciences, Columbia University, New York, NY, United States of America; 14 Department of Nutrition and Nutrition Research Institute, University of North Carolina, Chapel Hill, North Carolina, United States of America; Brigham and Women's Hospital and Harvard Medical School, UNITED STATES

## Abstract

**Background:**

Increased serum levels of C-reactive protein (CRP), an important component of the innate immune response, are associated with increased risk of cardiovascular disease (CVD). Multiple single nucleotide polymorphisms (SNP) have been identified which are associated with CRP levels, and Mendelian randomization studies have shown a positive association between SNPs increasing *CRP* expression and risk of colon cancer (but thus far not CVD). The effects of individual genetic variants often interact with the genetic background of a population and hence we sought to resolve the genetic determinants of serum CRP in a number of American Indian populations.

**Methods:**

The Strong Heart Family Study (SHFS) has serum CRP measurements from 2428 tribal members, recruited as large families from three regions of the United States. Microsatellite markers and MetaboChip defined SNP genotypes were incorporated into variance components, decomposition-based linkage and association analyses.

**Results:**

CRP levels exhibited significant heritability (h^2^ = 0.33 ± 0.05, p<1.3 X 10^−20^). A locus on chromosome (chr) 6, near marker D6S281 (approximately at 169.6 Mb, GRCh38/hg38) showed suggestive linkage (LOD = 1.9) to CRP levels. No individual SNPs were found associated with CRP levels after Bonferroni adjustment for multiple testing (threshold <7.77 x 10^−7^), however, we found nominal associations, many of which replicate previous findings at the *CRP*, *HNF1A* and 7 other loci. In addition, we report association of 46 SNPs located at 7 novel loci on chromosomes 2, 5, 6(2 loci), 9, 10 and 17, with an average of 15.3 Kb between SNPs and all with p-values less than 7.2 X 10^−4^.

**Conclusion:**

In agreement with evidence from other populations, these data show CRP serum levels are under considerable genetic influence; and include loci, such as near *CRP* and other genes, that replicate results from other ethnic groups. These findings also suggest possible novel loci on chr 6 and other chromosomes that warrant further investigation.

## Introduction

Immune and inflammatory factors have longstanding roles in microbial infection [1,2] and auto-immune disorders [3]; it is becoming increasingly clear that they also influence the pathogenesis and complications of metabolic conditions [4], cancer [5] and other chronic diseases [6]. C-reactive protein (CRP) is a prominent component of the innate immune system involved in non-self recognition and destruction [7] and has been employed as a non-specific measure of inflammatory status in epidemiologic and clinical studies of numerous disorders [8–10]. For example, elevated serum CRP is prospectively associated with a number of cancer types, including colon [11], breast [12] and lung [13]. In addition, two studies using Mendelian randomization approaches to assess the influence of inherited increases in CRP level on the risk for colorectal cancer, supported a causal relationship between increased CRP and cancer [14,15]. Evidence for a genetic influence on the relationship between immune factors, metabolic syndrome and cardiovascular risk factors is provided by the association of alleles increasing CRP levels and increased risk of obesity [16,17].

The interaction between genetic influences on basal CRP levels and a number of environmental factors has been investigated using heritability estimation [18], candidate gene [19], genome-wide linkage [18], genome-wide association (GWAS) [20,21] and other genetic approaches [22]. Some of the more compelling results from GWAS are summarized in Table 1. In general, excluding the *CRP* gene itself, 7 genomic regions of have been associated with CRP in these studies. Within 5 Mb of the *CRP* gene, variants of *IL6R* were shown to be independently associated to CRP levels [19]. Another chromosome (chr) 1 locus encompassing the *LEPR/JAK1* genes (which play a key role in immune response pathways [23]) has several SNPs associated with serum CRP [21,24]. On chr 2, SNPs at *GCKR* have been implicated in regulation of CRP expression [24]. SNPs near the *EPHA7* and *IL6* genes on chr 6 and 7 respectively, show association with CRP levels as well [25,26]. Variation in the *HNF1A* region of chr 12 has been repeatedly reported to be correlated with CRP expression [27,28]. Variants in three genes,*TOMM40*, *APOE* and *APOC1*, in a 30 Kb span of chr 19 are prominently related to serum CRP [19,24,25].

**Table 1:** GWAS Catalog [115] and selected SNP associations with serum CRP from the literature.

SNP	Chr	Coordinates	Risk Freq	β or Odds Ratio	P-value	Risk Allele	Upstream	Intragenic	Downstream	ethn*	Ref**
rs1805096	1	65,636,574	0.37	-0.11	3.6 X 10^−8^	G	No	LEPR	No	EUR	[72]
			0.46	-0.11	5.4 X 10^−5^					AA	[72]
rs1892534	1	65,640,261	0.39	-0.08	5.8 X 10^−8^	T	No	LEPR	No	EA	[24]
			0.46	-0.08	4.4 X 10^−3^					AA	[24]
rs4420065	1	65,695,778	0.39	0.09	3.5 X 10^−62^	C	LEPR	No	PDE4B	EA	[21]
rs4129267	1	154,453,788	0.40	-0.08	2.1 X 10^−48^	T	No	IL6R	No	EA	[21]
			0.40	-0.08	5.2 X 10^−21^					EA	[19]
			0.13	-0.12	5.7 X 10^−7^					AA	[19]
rs2228145	1	154,454,494	0.40	-0.12	7.8 X 10^−11^	C	No	IL6R	No	EUR	[72]
			0.14	-0.09	2.6 X 10^−2^					AA	[72]
rs12093699	1	159,678,198	0.29	NA***	6.0 X 10^−6^	NA	OR10J6P	No	CRPP1	EUR	[112]
rs10494326	1	159,679,910	0.178	0.4199	4.0 X 10^−73^	T	OR10J6P	No	CRPP1	AA/HIS	[20]
rs726640	1	159,685,728	NA	0.44	2.0 X 10^−13^	NA	OR10J6P	No	CRPP1	AA	[116]
rs2592902	1	159,685,936	0.38	NA	1.0 X 10^−9^	A	OR10J6P	No	CRPP1	EA/AA	[94]
rs12755606	1	159,700,546	NA	NA	4 X 10^−120^	C	OR10J6P	No	CRPP1	EUR	[117]
rs876537	1	159,705,143	0.43	0.29	1.4 X 10^−9^	C	No	CRPP1	No	FIL	[25]
rs16842559	1	159,706,381	0.89	0.106	4.0 X 10^−21^	T	CRPP1	No	CRP	AS	[118]
rs2794520	1	159,709,026	0.66	0.16	2 X 10^−186^	C	CRPP1	No	CRP	EUR	[21]
			0.34	NA	3.0 X 10^−8^					EA	[119]
			0.60	0.19	1.8 X 10^−15^					EA	[29]
			NA	-0.20	4.7 X 10^−26^					EA	[77]
rs1205	1	159,712,443	0.33	-0.17	1.0 X 10^−31^	T	No	CRP	No	EA	[24]
				-0.199	1.65 X 10^−26^					EA	[77]
			0.20	-0.27	8.1 X 10^−15^					AA	[24]
			0.35	-0.22	5.37 X 10^−09^					HIS	[24]
			0.46	-0.26	8.5 X 10^−09^					FIL	[25]
rs1800947	1	159,713,648	0.06	-0.30	3.1 X 10^−25^	G	No	CRP	No	EUR	[24]
			0.01	-0.61	1.3 X 10^−6^					AA	[24]
			0.02	-0.36	6.7 X 10^−3^					HIS	[24]
			0.06	-0.27	4.8 X 10^−12^					EUR	[72]
			0.01	-0.58	1.5 X 10^−5^					AA	[72]
rs77832441	1	159,714,024	0.002	-0.75	1.4 X 10^−4^	A	No	CRP	No	EUR	[72]
			0.005	-2.06	6.6 X 10^−4^					AA	[72]
rs1417938	1	159,714,396	0.30	0.14	5.6 X 10^−7^	A	No	CRP	No	EUR	[24]
			0.11	0.20	1.2 X 10^−2^					AA	[24]
			0.36	0.14	2.7 X 10^−4^					HIS	[24]
rs3091244	1	159,714,875	0.38	0.17	3.5 X 10^−91^	G	CRP	No	RPL27P2	EA	[19]
			0.55	0.24	5.1 X 10^−45^					AA	[19]
			0.08	0.26	5.2 X 10^−7^					FIL	[25]
			NA	0.20	6.0 X 10^−28^					EA	[77]
rs3093059	1	159,715,346	0.12	0.161	4.0 X 10^−21^	G	CRP	No	RPL27P2	JPT	[30]
rs3093058	1	159,715,525	0.001	0.32	1.4 X 10^−1^	T	CRP	No	RPL27P2	EUR	[24]
			0.17	0.48	1.4 X 10^−40^					AA	[24]
			0.01	0.67	1.0 X 10^−3^					HIS	[24]
rs1341665	1	159,721,769	0.96	-0.19	2.0 X 10^−20^	A	CRP	No	RPL27P2	EA	[29]
rs2808634	1	159,722,783	0.156	0.153	3.0 X 10^−10^	T	CRP	No	RPL27P2	EA/AA	[21]
rs7553007	1	159,728,759	NA	OR 20.7	8.0 X 10^−44^	NA	CRP	No	RPL27P2	EUR/AS	[28]
			0.344	0.129	1.0 X 10^−9^	G				HIS	[20]
			0.228	0.272	1.0 X 10^−37^	T				AA	[20]
			0.369	0.164	2.0 X 10^−16^	C				AS	[120]
			0.327	0.202	7.0 X 10^−12^	A				EUR	[121]
rs11265260	1	159,730,249	NA	NA	7.0 X 10^−6^	NA	CRP	No	RPL27P2	EUR/EA	[108]
rs7561273	2	24,024,644	0.35	0.22	6.0 X 10^−6^	A	No	MFSD2B	No	MIC	[124]
rs1260326	2	27,508,073	0.41	0.07	4.6 X 10^−40^	G	No	GCKR	No	EA	[21]
			0.42	0.10	2.4 X 10^−17^					EA	[19]
			0.15	0.06	2.1 X 10^−2^					AA	[19]
				0.03	1.0 X 10^−3^					AS	[26]
rs780094	2	27,518,370	0.40	0.10	1.5 X 10^−16^	C	No	GCKR	No	EA	[24]
			0.19	0.03	2.3 X 10^−1^					AA	[24]
			0.34	0.07	2.6 X 10^−2^					HIS	[24]
rs1441169	2	213,168,806	0.53	-0.03	2.3 X 10^−11^	G	LINCO1953	No	IKZF2	EUR	[64]
rs960246	2	223,072,841	0.013	0.22	1.0 X 10^−9^	T	KCNE4	No	LOC105373905	AS	[122]
rs1514895	3	170,987,904	0.71	-0.03	2.7 X 10^−9^	A	SLC2A2	No	EIF5A2	EUR	[86]
rs16871289	4	21,509,760	0.017	0.03	9.0 X 10^−6^	A	No	KCNIP4	No	HIS	[123]
rs6846071	4	101,481,058	0.016	0.224	1.0 X 10^−11^	G	FLJ20021	No	LOC105377346	AS	[122]
rs283610	5	73,952,687	0.456	0.03	7.0 X 10^−6^	G	ARHGEF28	No	CTD-2292M14.1		[123]
rs465384	5	125,907,327	NA	NA	1.0 X 10^−6^	NA	RP11-756H20.1	No	RP11-114J13.1	EUR	[117]
rs17658229	5	172,764,049	0.05	0.06	5.5 X 10^−9^	C	AC022217.2	No	DUSP1	EUR	[86]
rs1408282	6	93,142,534	0.10	0.37	2.9 X 10^−6^	A	COPS5P1	No	EPHA7	FIL	[25]
rs6904416	6	98,542,613	0.019	0.183	9.0 X 10^−10^	C	RP11-436D23.1	No	POU3F2	AS	[122]
rs12202641	6	115,993,471	0.39	-0.02	3.0 X 10^−10^		No	FRK	No	EUR	[86]
rs9385532	6	130,050,082	0.33	-0.03	1.9 X 10^−11^		No	L3MBTL	No	EUR	[86]
rs6907728	6	131,907,629	0.186	0.04	3.0 X 10^−6^	C	ENPP1	No	CTGF	HIS	[123]
rs2097677	7	22,693,220	NA	0.05	2.6 X 10^−9^	A	AC002480.2	No	IL6	AS	[26]
rs1880241	7	22,719,850	0.48	-0.03	8.4 X 10^−14^	G		No	IL6	EUR	[86]
rs2710804	7	36,044,919	0.37	0.02	1.3 X 10^−8^	C	lncRNA	No	EEPD1	EUR	[86]
rs6956675	7	63,117,392	0.135	0.03	6.0 X 10^−6^	A	SAPCD2P4	No	SEPT14P1	HIS	[123]
rs10255299	7	111,887,504	0.013	0.241	7.0 X 10^−11^	G	No	DOCK4	No	AS	[122]
rs10125337	9	94,681,756	0.004	0.03	4.0 X 10^−6^	G	FBP1	No	LOC107987101	HIS	[123]
rs643434	9	133,266,943	0.37	0.02	1.0 X 10^−9^	A	No	ABO	No	EUR	[86]
rs7076247	10	18,470,700	0.37	NA	6.0 X 10^−6^	NA	No	CACNB2	No	EUR	[112]
rs11066587	12	113,541,851	0.16	0.26	5.0 X 10^−6^	G	LHX5-AS1	No	LOC105369990	MIC	[124]
rs1039302	12	120,798,455	0.36	0.21	5.0 X 10^−6^	T	No	SPPL3	No	MIC	[124]
rs2650000	12	120,951,159		-0.12	7.1 X 10^−11^	A	No	HNF1A-AS1	No	EA	[77]
			0.35	-0.12	2.6 X 10^−23^					EA	[24]
			0.12	-0.09	5.2 X 10^−3^					AA	[24]
			0.36	-0.11	9.5 X 10^−4^					HIS	[24]
rs7305618	12	120,965,129	0.52	0.267	1.0 X 10^−8^	T	No	HNF1A-AS1	No	FIL	[25]
rs7953249	12	120,965,921		-0.13	7.0 X 10^−13^	G	No	HNF1A-AS1	No	EA	[77]
rs1169289	12	120,978,819	0.46	-0.12	9.0 X 10^−11^	G	No	HNF1A	No	EUR	[72]
			0.34	-0.06	2.2 X 10^−2^					AA	[72]
rs1169288	12	120,978,847	0.34	-0.11	9.5 X 10^−9^	C	No	HNF1A	No	EUR	[72]
			0.12	-0.08	4.0 X 10^−2^					AA	[72]
rs1183910	12	120,983,004	0.31	0.11****	6 X 10^−76^	A	No	HNF1A	No	EA	[27]
				14%*****	1.2 X 10^−17^					EA	[28]
			0.33	-0.15	2 X 10^−124^					EA	[21]
rs2393791	12	120,986,153	0.478	0.049	3.0 X 10^−9^	C	No	HNF1A	No	AS	[122]
rs7310409	12	120,987,058	0.40	-0.18	1.6 X 10^−10^	A/G	No	HNF1A	No	EA	[24]
			0.32	-0.15	7.9 X 10^−3^					AA	[24]
			0.41	-0.13	1.1 X 10^−3^					HIS	[24]
			0.53	-0.07	2.7 X 10^−8^					JPT	[30]
			0.67	-0.22	1.6 X 10^−6^					FIL	[25]
rs2259820	12	120,997,539	0.31	-0.12	1.8 X 10^−9^	T	No	HNF1A	No	EUR	[72]
			0.12	-0.07	9.6 X 10^−2^					AA	[72]
rs2464196	12	120,997,624	0.32	-0.12	9.3 X 10^−9^	A	No	HNF1A	No	EUR	[72]
			0.12	-0.07	8.1 X 10^−2^					AA	[72]
rs1169310	12	121,001,630	0.38	0.13	2.0 X 10^−8^	A	No	HNF1A	No	EUR	[108]
rs2526932	14	72,614,360	0.012	0.275	6.0 X 10^−13^	G	RP3-514A23.2	No	DPF3	AS	[122]
rs2239222	14	72,545,177	0.36	0.04	9.9 X 10^−20^	G	No	RGS6	No	EUR	[86]
rs112635299	14	94,371,805	0.02	-0.11	2.1 X 10^−10^	T	SERPINA1	No	SERPINA2	EUR	[86]
rs178810	17	16,194,116	0.56	0.02	2.9 X 10^−8^	T	No	NCOR1	No	EUR	[86]
rs892073	19	29,421,387	0.044	0.03	8.0 X 10^−6^	A	No	CTC-525D6.1	No	HIS	[123]
rs2075650	19	44,892,362	0.15	-0.12	4.2 X 10^−8^	G	No	TOMM40	No	EA	[65]
			0.14	-0.22	1.8 X 10^−38^					EA	[24]
				-0.21	6.8 X 10^−16^					EA	[77]
			0.13	-0.18	2.2 X 10^−47^					EA	[19]
			0.14	-0.02	6.5 X 10^−1^					AA	[24]
rs157581	19	44,892,457	0.21	-0.16	2.4 X 10^−12^	C	No	TOMM40	No	EUR	[72]
			0.47	-0.09	5.1 X 10^−4^					AA	[72]
rs11556505	19	44,892,887	0.14	-0.18	2.9 X 10^−11^	T	No	TOMM40	No	EUR	[72]
			0.12	-0.02	6.3 X 10^−1^					AA	[72]
rs112849259	19	44,894,050	0.03	-0.28	1.3 X 10^−6^	C	No	TOMM40	No	EUR	[72]
			0.04	-0.37	1.1 X 10^−7^					AA	[72]
rs769449	19	44,906,745		0.38	6.8 X 10^−3^	G	No	APOE	No	FIL	[125]
rs769450	19	44,907,187		0.03	1.0 X 10^−4^	NA	No	APOE	No	EA	[19]
			0.37	0.08	1.6 X 10^−6^					AA	[19]
rs429358	19	44,908,684	0.11	-0.31	7.0 X 10^−8^	C	No	APOE	No	EUR	[72]
			0.19	-0.24	1.5 X 10^−6^					AA	[72]
rs4420638	19	44,919,689	0.18	-0.24	1.0 X 10^−56^	G	APOC1	No	APOC4	EA	[24]
			0.20	-0.03	2.7 X 10^−1^					AA	[24]
			0.10	-0.18	5.6 X 10^−4^					HIS	[24]
			0.20	-0.24	9 X 10^−139^					EA	[21]
			0.21	-0.28	1.6 X 10^−6^					MIC	[124]
rs2159324	19	45,192,480	0.44	0.19	2.0 X 10^−6^	T	No	AC005779.2	No	MIC	[124]
rs2315008	20	63,712,604	0.31	-0.02	5.4 X 10^−10^	T	No	ZGPAT	No	EUR	[86]
rs2315656	20	63,786,984	0.395	0.03	4.0 X 10^−6^	G	No	ZBTB46	No	HIS	[123]

* ethnicity, EA: European American, AA: African American, HIS: Hispanic, FIL: Filipino, AS: Asian, JPT: Japanese, MIC: Micronesia, EUR: European.

** endnote reference.

*** NA: not available.

**** per allele effect in z score units -0.11 (lnCRP).

***** % change in ln CRP per minor allele.

Although genetic variants common across populations associate with CRP levels, there also appear to be variants in multiple loci across the genome with differential strength of effects [24,29,30], or that are found primarily in certain populations, such as African Americans [19], and in some cases very restricted in prevalence (Aboriginal Canadians [31]). There have been very few studies focused on indigenous populations of North and South America [31–33]; and none employing linkage or GWAS analysis. These have shown similar heritability of CRP (29 to 46%) and differing population prevalences of variants affecting CRP levels.

Cardiovascular disease (CVD) [34], diabetes mellitus (DM) [35] and other conditions [36] with a significant inflammatory component account for a disproportionately large fraction of mortality and morbidity in American Indian (AI) communities. A better understanding of the genetic contributions to this important component of the innate immune system may shed light on some of these health disparities. Unfortunately genetic research among indigenous peoples has become more challenging after the inappropriate activities of some investigations have been revealed [37,38]. The resulting lack of trust in investigators, exhibited among AI and other populations can have many important societal impacts, including the possibility of worsening already adverse health disparities [39,40]. The aim of this study is to identify genetic loci influencing basal CRP levels using genome-wide linkage and extensive SNP genotyping among participants in a large and well-characterized cohort of American Indians, the Strong Heart Family Study (SHFS).

## Methods

### Population

The Strong Heart Study (SHS) is a population-based, cohort study of CVD and associated risk factors among American Indians in three centers in Arizona, Oklahoma and North/South Dakota. The participating communities, study design, survey methods and laboratory techniques have been described previously [41,42]. The SHS was extended in 1998 and subsequent phases, as the Strong Heart Family Study, recruiting participants 16 years and older, without regard to disease status, from multi-generational families, including index members of the SHS cohort. All participants have given written, informed consent. In addition, approval for this study was obtained from relevant tribal communities and institutional review boards, including Great Plains Indian Health Service (IHS) Institutional Review Board (IRB), Oglala Sioux Research Review Board, Oklahoma IHS IRB, University of Oklahoma IRB, Phoenix Area IHS IRB, MedStar Health Research Institute IRB, University of North Carolina IRB, Columbia University IRB, and University of Texas Health IRB. The collection of phenotypic data for the SHFS was conducted between 2001 and 2003 according to methods described previously [41]. "Ever" smoking was defined as having smoked at least 100 cigarettes during the lifetime and "current" smoking as present, regular use of smoke tobacco. "Current" and "ever" alcohol intake was defined as having had at least 12 alcoholic beverages in the last year or in past years, respectively.

### Biomarker, serum CRP

CRP was measured using a immunoturbidometric method (Vitros Chemistry Products, number 6801739, Ortho Clinical Diagnostics, Rochester, NY), on a Vitros 5,1 platform (Ortho Clinical Diagnostics, Rochester, NY). This method has shown good comparability to results from the previous Dade-Behring immunonephelometric method [43].

### Genome-wide linkage analysis, quality control

The procedures for genotyping microsatellite markers in the SHFS have been described previously [44]. In brief, DNA was amplified with primers specific for short tandem repeat markers using the ABI PRISM Linkage Mapping Set-MD10 Version 2.5 (Applied Biosystems, Foster City, CA). PCR products were loaded into an ABI PRISM 377 DNA sequencer for laser-based automated genotyping. Analyses and assignment of the marker alleles were done using computerized algorithms (Applied Biosystems). deCODE Genetics provided sex-averaged chromosomal maps (in units of Haldane centi-morgans) for this analysis [45]. Pedigrees were screened with the Pedigree Relationship Statistical Tests (PREST) [46] and SimWalk2 [47] programs for checking for Mendelian inconsistencies and possible double recombinants. The above screening resulted in less than 1% of all genotypes being excluded. Multipoint identity-by-descent (IBD) matrices for genome-wide linkage analyses were calculated using the linkage analysis package LOKI [48].

### Genome-wide association analysis, quality control

To study potential effects of environmental exposures on incident diabetes [49], a subset of SFHS [42] without prevalent diabetes has been genotyped utilizing the Illumina Human Cardio-Metabo BeadChip array (MetaboChip, Illumina, San Diego, CA), an Illumina custom panel incorporated 196,725 SNPs previously identified as significant GWAS signals for metabolic and CVD traits [50]. Blood samples collected from individuals who were free of DM at baseline visit were used for this study and genotyped at the Texas Biomedical Research Institute, San Antonio, TX. All genomic positions listed are derived from NCBI GRCh38/hg38.

Non-autosomal (n = 250) and monomorphic markers (n = 158) were removed prior to genotyping quality control. Mendelian inconsistencies were excluded using Preswalk, a PEDSYS compatible version of Simwalk2 [47]. SNPs with a marker call rate < 98% or no data (n = 33,604) and individual samples with a call rate < 95% (n = 3) were excluded. Allele frequency and Hardy-Weinberg equilibrium (HWE) values were estimated using Sequential Oligogenic Linkage Analysis Routines (SOLAR) [51]. Markers failing HWE analysis at p < 10^−5^ (n = 1,519) and those with minor allele frequencies (MAF) less than 1% (n = 40,219) were also excluded. Since there have been reports of duplicate sequences surrounding certain SNPs (most easily recognized when the duplicate is on a sex chromosome) [52], we conducted an additional screen for significant differences in genotype distribution between genders among the 69 SNPs with association p-values <4X10^-4^ and passing the previously described, typical screens. Within this group there were 3 SNPs that showed significant differences (p<0.05) in genotype and allelic distribution between genders and were thus excluded. Details from two examples are presented in the S1 Table.

Pairwise correlations (r^2^) between markers were calculated to estimate linkage disequilibrium (LD). The original annotation file for the Cardio-Metabo BeadChip, “MetaboChip_Gene_Annotation” is accessible through the Illumina website. A PEDSYS [53] compatible version of Merlin [54] was used for pedigree-guided imputation of array marker data using the UCSC Genome Browser hg18 assembly [55]. The lack of comparable data sources for AI populations necessitated the use of primarily European data from the UCSC assembly. The final data set includes 120,972 autosomal markers with information available for MetaboChip analysis of 1,892 AI participants.

### Statistical analysis

#### Genome-wide linkage analysis

We used stepwise linear regression in center stratified samples to screen covariates (SAS, *version 8*.*0*). Quantitative genetic analysis was conducted using a maximum likelihood variance components decomposition-based method [51]. This approach was implemented in the computer program SOLAR, version 8.1.1 [51] which allows for an explicit test of whether phenotypic covariance among family members are in part due to genetic effects.

A total of 2,428 SHFS participants were considered for linkage analysis (Arizona (AZ) = 286 Dakota (DK) = 1,066, Oklahoma (OK) = 1,076), as seen in Table 2, after excluding those individuals with missing covariate data and as indicated below to normalize the phenotypic trait distribution. Because variance components methods are sensitive to kurtosis [56] and to avoid including those with an acute inflammatory process, phenotypic outliers (N = 195) with CRP levels >16.0 (~3 standard deviations (SD) above the mean) were removed prior to analysis. In addition, CRP levels were natural log transformed. All analyses were conducted separately for each center and then on the combined data from all three centers. To maximize our power to detect genetic effects, a minimally adjusted model (Model 2, Table 3), incorporating age, age^2^, age*sex, age^2^*sex, sex, and center covariates was analyzed first. Secondary analyses considered adjustment for the linear fixed effects of the covariates listed in Table 2, which were previously shown to influence the trait in epidemiological studies [32,57–59]. We additionally confirmed the significance of Model 2 covariates while accounting for family relationships in SOLAR. Residuals were generated for Model 2 and used in all subsequent genetic analyses. Kurtosis values for CRP were < 0.50 for all analyses.

**Table 2:** Descriptive characteristics of SHFS participants stratified by study recruitment center.*

	Linkage Study			SNP Association Study		
	AZ	DK	OK	AZ	DK	OK
Participants (N)	286	1066	1076	195	901	796
Gender (female)	66.4%	58.7%	57.2%	65.5%	60.0%	58.6%
Age, years mean (± SD)	37.7 (16.6)	38.5 (16.8)	43.3 (17.3)	33.2 (14.6)	36.5 (15.9)	40.2 (16.1)
Pedigrees, N	13	16	9			
Generations	5	5	6			
(ln)hsCRP, in mg/L, mean (± SD)	1.267 (1.059)	0.964 (1.109)	0.976 (1.074)	1.196 (1.044)	0.904 (1.144)	0.896 (1.092)
Diabetes** N (%)	82 (29)	140 (13)	207 (19)	4 (9)	17 (2)	24 (3)
Smoking,*** ever/current, N (%)	133 (47)	701 (66)	625 (58)	71 (41)	556 (65)	430 (56)
Waist, cm, mean (± SD)	109.5 (20)	99.0 (17)	101.5 (17)	107.6 (21.4)	97.7 (16.2)	99.7 (16.6)
Menopausal Yes, N (%)****	50 (26)	157 (25)	224 (36)	16 (14)	105 (20)	127 (28)
Alcohol Current, N (%)	177 (62)	715 (67)	518 (48)	111 (65)	598 (70)	403 (52)
Total cholesterol mg/dl, mean (± SD)	173.6 (33.1)	181.7 (36.6)	185.8 (37.1)	171.9 (32.4)	181.0 (36.0)	184.7 (35.8)
HDL-Cholest, mg/dl mean (± SD)	49.3 (14.3)	50.7 (13.7)	52.9 (15.5)	48.8 (14.3)	50.8 (13.8)	54.0 (15.7)
LDL-Cholest, mg/dl mean (± SD)	94.2 (25.8)	100.9 (30.8)	100.1 (30.2)	94.6 (25.5)	101.1 (30.6)	100.1 (30.6)
Triglycerides, mg/dl mean (± SD)	158.2 (111.0)	157.3 (138.9)	172.1 (176.4)	147.8 (85.1)	150.9 (129.0)	157.1 (103.6)
Estrogen use Yes, N (%)	40 (21)	102 (16)	92 (15)	2 (2)	40 (8)	60 (13)
BMI, Kg/m^2^ mean (± SD)	34.4 (8.5)	29.9 (6.6)	30.9 (6.8)	34.1 (9.0)	29.8 (6.6)	30.4 (6.8)
HbA1c (%, ± SD)	7.0 (2.2)	6.0 (1.6)	6.5 (1.9)	5.7 (1.3)	5.5 (0.9)	5.6 (0.9)
Systolic blood pressure (mmHg)	119.7 (14.8)	119.9 (16.0)	126.4 (17.2)	117.3 (13.0)	118.5 (14.6)	124.2 (16.2)

* Percentages and means calculated only from those with available measurements.

** Diabetes was determined using the American Diabetes Association criteria.

*** Smoking was defined as "current" or "ever" smokers.

**** Percentage of females.

**Table 3:** Overall heritability assuming various models.

Model	Covariates included in the final model	h^2^ (SE)	P-value	Chrom (loc)	LOD score
1	None	0.29 (0.04)	4.x 10–17	5 (25)	0.074
				6 (185)	0.800
				6 (189)	1.037
				6 (191)	1.065
				19 (93)	1.611
2	age, age^2^, age x sex, age^2^ x sex, sex, center	0.33 (0.05)	1.3 x 10–20	5 (25)	0.002
				6 (185)	1.615
				6 (189)	1.825
				6 (191)	1.824
				19 (93)	1.431
3	age, age^2^, age x sex, age^2^ x sex, sex, center, smoking status	0.33 (0.05)	6.7 x 10–21	5 (25)	0.002
				6 (185)	1.583
				6 (189)	1.880
				6 (191)	1.896
				19 (93)	1.315
4	age, age^2^, age x sex, age^2^ x sex, sex, center, Waist circumference, Body fat, total cholesterol, triglycerides, HDL, LDL, HbA1C, Systolic blood pressure	0.32 (0.09)	9.7 x 10–6	5 (25)	2.002
				6 (185)	0.012
				6 (190)*	0.000
				6 (190)	0.000
				19 (90)	0.192
5	age, age^2^, age x sex, age^2^ x sex, sex, center, DM status	0.32 (0.05)	1.2 x10-18	5 (25)	0.002
				6 (185)	1.512
				6 (189)	1.807
				6 (191)	1.828
				19 (93)	1.463
6	age, age^2^, age x sex, age^2^ x sex, sex, center, hypertension status	0.33 (0.05)	1.3 x 10–19	5 (25)	0.000
				6 (185)	1.493
				6 (189)	1.714
				6 (191)	1.698
				19 (93)	1.332
7	age, age^2^, age x sex, age^2^ x sex, sex, center, hormone replacement therapy status	0.35 (0.07)	2.2 x 10–9	5 (25)	0.000
				6 (185)	1.561
				6 (189)	1.236
				6 (191)	1.002
				19 (95)	0.009
Highest LOD score in each center, compared with other centers					
Center	Covariates included in the final model	h^2^ (SE)	P-value	Chrom (loc)	LOD score
AZ	age, age^2^, age x sex, age^2^ x sex, sex	0.70 (0.16)	3.3 x 10^−6^	18 (36)	**2.360**
DK	age, age^2^, age x sex, age^2^ x sex, sex			18 (35)	0.001
OK	age, age^2^, age x sex, age^2^ x sex, sex			18 (35)	0.000
AZ	age, age^2^, age x sex, age^2^ x sex, sex			16 (50)	0.000
DK	age, age^2^, age x sex, age^2^ x sex, sex	0.33 (0.06)	3.2 x 10^−11^	16 (51)	**2.236**
OK	age, age^2^, age x sex, age^2^ x sex, sex			16 (50)	0.000
AZ	age, age^2^, age x sex, age^2^ x sex, sex			6 (190)	0.000
DK	age, age^2^, age x sex, age^2^ x sex, sex			6 (193)	0.499
OK	age, age^2^, age x sex, age^2^ x sex, sex	0.28 (0.07)	2.0 x 10^−7^	6 (193)	**1.284**

* closest available locus.

#### Genome-wide association analysis using MetaboChip array

MetaboChip genotyping was limited to the subset of SHFS without DM during the pilot (1997–1999) and the next phase (2001–2003), thus a total of 1,892 SHFS participants were included in the SNP association analysis. Linear regression models for CRP with each SNP were used under the assumption of an additive genetic model and the analysis was performed using variance components decomposition-based models to account for familial correlation, as implemented in the SOLAR software package [51]. This approach allows us to account for the non-independence among family members.

Principal component analysis (PCA) was used to derive principal component scores (PCs) modeling differences in ancestral contributions among study participants. PCs were calculated using the unrelated SHFS founders (n = 644) and a subset of 15,158 selected SNPs (r^2^ <0.1; MAF >0.05). PCA was performed on a matrix of “doses” (copies of minor allele) for the selected SNPs, using “prcomp” in R. The PCs were then predicted for all genotyped individuals using the PCA model fit to the founder data [60]. While no PC accounted for a large percentage of total variance in genotype scores, the first four PCs account for substantially more than the rest and were, therefore, included as additional covariates in association analyses.

To minimize the problem of non-normality, the CRP data were log-transformed. All analyses involved adjustment for basic covariates (age, age^2^, age*sex, age^2^*sex, sex^2^ and PCs). We stratified the association analysis by geographical location (Arizona, Oklahoma, North and South Dakota) to account for possible differences between the three locations. After consideration of linkage disequilibrium effects using the Moskvina and Schmidt method [61] the 120,972 analyzed SNPs had an effective size of 64,375 and the Bonferroni significance level was determined to be p <7.77 x 10^−7^. When considering SNPs or gene regions previously shown to be significantly associated with CRP in the literature, a p-value of 0.05 was considered evidence of replication.

#### Metal

METAL software [62] was used to perform meta-analysis of GWAS results taken from the three study centers, each study containing individual genome-wide MetaboChip association results for multiple markers are analyzed across all studies for marker(s) with significant results. The fixed-effect meta-analysis across the center-specific association results used I^2^ to assess heterogeneity across centers.

#### Additional analysis

We used fine mapping and conditional analysis to identify independent SNP associations within loci for CRP. We focused on chromosomal regions of 1, 12 and 19 (included in Table 1) due to better coverage of SNPs in these regions. For SNPs identified in the literature but not available on the MetaboChip, we used a strategy of examining all SNPs within a 1 Mb span of the published SNP, given that LD in AI is not available. Conditional association analysis was then conducted, using the proxy SNPs as covariates. The p-value for the hypothesis that a newly identified, secondary independent association exists, was calculated as 0.05 divided by the number of independent SNPs in the region.

## Results

The descriptive characteristics of CRP and other covariates, stratified by recruitment center, are displayed in Table 2. Women exhibited significantly higher mean CRP than men (3.64 +/- 2.84 mg/dl vs 2.27 mg/dl +/- 2.90). The CRP levels were highest (3.61 mg/dl ° 2.74) in the AZ center and lowest (2.58 mg/dl ° 3.01) in the DK center. Individuals from the AZ center had the highest prevalence of DM and obesity compared to the other centers. In contrast, SHFS participants from the DK center had the highest prevalence of current smokers. The OK center had the highest prevalence of women with menopause. The descriptive characteristics of the subsample with genotypes (N = 1,892) included in the MetaboChip analysis are given in Table 2. Essentially all of the MetaboChip cohort (99.6%) were included in the linkage analysis; but due to the exclusion of those with DM, only 70.1% of the linkage cohort were included in the MetaboChip analysis.

### Genome-wide linkage analysis of CRP

To estimate the proportion of the CRP level variance due to genetic effects (heritability), we used the full SHFS population. Heritability was significant for lnCRP levels (h^2^ = 0.33 ± 0.05 with adjustment for demographic covariates in model 2, p<1.3 X 10^−20^) (Table 3). Further adjustment for smoking (model 3), measures of obesity and serum lipid levels (model 4), DM (model 5), or hypertension (model 6) provided similar estimates. In center stratified analyses, heritability estimates are shown in Table 3, with the highest at 0.71 ± 0.16 in the Arizona center.

Using the best heritability model (model 3) we next examined the evidence of linkage for lnCRP. Logarithm of the odds (LOD) scores >1.9 are generally accepted as suggestive genome-wide, multipoint linkage [63]. Thus there was suggestive evidence (LOD = 1.90) from model 3 for linkage of CRP to a locus on chr 6, 191cM, near marker D6S281, corresponding to a physical position at approximately 169.6 Mb (GRCh38/hg38). The signal was maximal with these typical adjustments for smoking and demographic covariates; and was only slightly attenuated after inclusion of DM (LOD = 1.83) and hypertension (LOD = 1.71) in the model. Adjustment of model 2 for measures of adiposity, systolic blood pressure and serum lipids, markedly attenuated the signal (LOD = 0.41) at this position. The LOD score was reduced in each center-specific analyses at this locus, with the highest being 1.36 for model 2 in the Dakota center and 1.28 for the OK center. The AZ center failed to show any sign of linkage at this locus; but was hindered by a small sample size at that center.

At chr 16, the strongest linkage signal was found with model 2 at 51cM (D16S3068) with a LOD score of 2.24 in the DK center. At this locus, however, the other centers show virtually no signal, suggestive of a population-specific association. No individual center showed any noteworthy signals on chr 19, with the maximum being 1.09 in the DK center. A maximum LOD score of 2.36 was noted in the AZ center on chr 18 at 36cM; but there was no corresponding signal in the DK and OK centers, with the maximal LOD score of 1.30 in the DK center observed at 52cM.

### Genome-wide analysis of SNPs for association with CRP

The main findings from association analyses using the MetaboChip genotyping data and standard covariates (age, age^2^, age*sex, age^2^*sex, sex^2^ and PCs) are summarized in Table 4 (restricted to those with a p-value of less than <7.0 x 10^−5^). Considering all three centers together, no SNPs demonstrated a Bonferroni-corrected, MetaboChip wide, statistically significant association (p-value < 7.77 x 10^−7^). It should be noted that in the interest of conserving space, there were 2 additional SNPs at the PHACTR1 locus, two at TARID, one at RP1L1, one at TCF7L2 and two at HNF1A, also with association p-values less than 7.0 x 10^−5^.

**Table 4:** MetaboChip results, combined center analysis, by chromosome, with association p-values (maximum of 7.0 X 10^−5^).

SNP	Gene*	Chr:	Min/maj allele**	z-score***	P	Physical Coordinates****	MAF
rs7595184	CENTG2	2	A/G	4.01	6.2 X 10^−5^	235,596,138	0.04
rs7617596	FOXP1	3	A/G	-4.03	5.6 X 10^−5^	71,472,343	0.329
rs1127343	C3orf28	3	A/G	4.54	5.6 X 10^−6^	122,409,547	0.147
rs7635320	MDS1	3	A/G	-3.99	6.7 X 10^−5^	169,246,830	0.356
rs4583704	ZNF509	4	C/G	4.03	5.5 X 10^−5^	4,372,749	0.486
rs200200	FARS2	6	A/G	4.34	1.4 X 10^−5^	5,443,407	0.03
rs9472752	PHACTR1	6	A/G	4.01	6.1 X 10^−5^	12,863,902	0.383
rs7740975	SLC35F1	6	A/G	4.24	2.2 X 10^−5^	118,158,542	0.112
rs1966248	TARID	6	A/T	-4.07	4.8 X 10^−5^	133,838,484	0.342
rs1294948	RAMP3	7	A/C	-4.2	2.7 X 10^−5^	45,172,191	0.216
rs7814795	RP1L1	8	A/G	-4.4	1.1 X 10^−5^	10,661,775	0.324
rs2577888	YTHDF3	8	A/C	4.08	4.5 X 10^−5^	64,364,912	0.257
rs10739202	PTPRD	9	A/G	-4	6.2 X 10^−5^	9,897,289	0.013
rs10733682	LMX1B	9	A/G	4.81	1.5 X 10^−6^	126,698,635	0.243
rs4132670	TCF7L2	10	A/G	-4.06	5.0 X 10^−5^	113,008,012	0.108
rs2393791	HNF1A	12	A/G	4.59	4.5 X 10^−6^	120,986,153	0.409
rs927791	FGF9	13	A/G	4.03	5.6 X 10^−5^	21,734,552	0.114
rs4531650	EGLN3	14	C/G	4.07	4.7 X 10^−5^	34,047,374	0.478
rs7143416	SLC24A4	14	A/G	4.23	2.4 X 10^−5^	92,452,449	0.284
rs3794808	SLC6A4	17	A/G	4.13	3.7 X 10^−5^	30,204,775	0.385
rs9902290	SNIP	17	C/G	3.98	7.0 X 10^−5^	38,651,325	0.044
**Center specific analysis**							
**SNP**	**Gene**	**Chr:**	**Min/maj** **allele**	**β**	**P**	**Physical** **coordinates**	**MAF**
**Arizona**							
rs1877715	CXCR1	2	A/G	-1.02	1.2 X 10^−6^	218,187,823	0.07
rs704951	XYLB	3	G/A	-1.36	3.3 X 10^−8^	38,372,036	0.06
rs12356821	WBP1L	10	C/G	-1.93	1.1 X 10^−8^	102,804,051	0.03
rs11195703	ncRNA	10	G/A	-0.73	3.6 X 10^−6^	111,741,277	0.13
rs72858840	SBF2	11	A/C	-2.15	5.6 X 10^−7^	10,033,699	0.02
rs12939525	CEP131	17	G/A	-1.15	1.2 X 10^−6^	81,220,205	0.07
**Dakota**							
rs12735411	ATP1A1	1	C/T	-0.48	1.3 X 10^−5^	116,353,438	0.07
rs1205	CRP	**1**	A/G	-0.23	2.4 X 10^−5^	159,712,443	0.52
rs1341665	CRP	1	A/G	0.24	1.6 X 10^−5^	159,721,769	0.47
rs11986935	PINX1	8	A/T	-0.27	4.4 X 10^−5^	10,834,039	0.25
rs1328648	DCLK1	13	A/G	0.23	3.2 X 10^−5^	36,148,879	0.43
rs74876483	SKOR1	15	C/T	0.34	2.0 X 10^−5^	67,861,793	0.14
**Oklahoma**							
rs4895389	TARID	6	C/T	-0.28	2.0 X 10^−6^	133,838,014	0.34
rs1969783	TARID	6	C/T	-0.28	2.6 X 10^−6^	133,838,261	0.35
rs1966248	TARID	6	A/T	-0.29	1.3 X 10^−6^	133,838,484	0.34
rs231350	KCNQ1	11	A/C	-0.26	5.5 X 10^−6^	2,692,419	0.39
rs2014429	PAUPAR	11	A/G/T	0.26	8.7 X 10^−6^	31,919,489	0.39

* Most proximal candidate gene.

** Minor allele is effect allele, major is referent.

*** inverse weighted average of three centers.

**** GRCh38.p7, dbSNP build 1.

These results corroborate previous literature reports of association between CRP and the SNPs in Table 1. Of the 77 variants previously linked to CRP, 27 were also identified as having similar associations in the present study, such as 4 SNPs in the *CRP* region, all with p-values less than 9 x 10^−4^, and 10 SNPs at *HNF1A* with p-values ranging from 0.07 to 4.4 x 10^−6^ for rs2393791. Of interest, a recent meta-analysis of over 200,000 Europeans found 58 novel variants [64], including rs178810 on chr 17, significantly associated with CRP, which was replicated by the current study (p-value 0.038). At the previously reported chr 19 region spanning *APOE*, *TOMM40* and *APOC1* [19,65], there were 3 SNPs with duplicate genotyping in the current study, all with nominal significance and direction of effect concordant with the literature [19,65].

We found two additional, nominally significant SNPs relatively proximal to others reported associated with CRP level, such as rs7635320 within 1.5 Mb of reported rs1514895 on chr 3, and rs7143416 within 2 Mb of reported rs112635299 on chr 14.

Alternatively, when the available MetaboChip SNPs were searched by region for the genes listed in Table 1, there are 3 SNPs intronic to *JAK1* (and approximately 400 Kb from the reported *LEPR* gene) on chr 1, all with association p-values less than 4.5 x 10^−3^. Also on chromosome 1, we found 5 SNPs in the IL6R region with association p-values less than 0.05. At the GCKR locus of chromosome 2, seven SNPs were identified of nominal significance. The above mentioned meta-analysis also found 2 additional variants (implicating FRK and ABO on chr 6 and 9) [64] which have 7 and 4 SNPs represented in the present analysis, all with p-values less than 0.05.

S2 Table highlights 3 clusters of nominally significant SNPs co-localizing with the CRP, HFN1A, and TOMM40/APOE/APOC1 regions, which include five SNPs within a 39Kb region, 19 SNPs within 43 Kb, and 6 SNPs within 27 Kb respectively. In addition, another 7 compact chromosomal regions are shown (S1 Table), suggesting that these SNPs are in linkage disequilibrium, potentially with a functional variant. These include a cluster of 6 SNPs within 28 Kb on chr 2, and a cluster of 5 SNPs within a 194 Kb region of chr 5. The latter group is within ~1 Mb of a novel SNP recently reported associated with CRP [64]. On chr 6 there are two groups, one of 12 SNPs within an 148 Kb region (centered at 12,825,000) and one of 5 SNPs within 55Kb (centered at 133,850,000). The latter is again within 2 and 4 Mb from two previous literature reports of associated SNPs. A group of 5 SNPs clustered within 10 Kb is found on chr 9, within ~200 Kb of a newly reported SNP [64]. There are 10 SNPs centered around 113,120,000 and within 243 Kb on chr 10, and another 3 SNPs reside within a 17 Kb area of chr 17. The SNPs in these clusters range from 1: 1,800bp to 1:39,000bp; and except for the chr 19 cluster, all of the p-values are <1x10^-3^. Please see figures S1–S10 Figs, showing locus zoom (A) and linkage disequilibrium plots (B) for each of these clusters in regions of chromosomes 1,2,5,6a/b,9,10,12,17 and 19.

Center specific analysis (Table 4, last section) revealed considerable differences between centers in strength of association among SNPs. For example, the OK center showed consistently strong association (all p-values less than 2.6 x 10^−6^) for three chr 6 SNPs tightly clustered within only 470 bp of each other; and within an intron of the *TARID* gene, which also encompasses the chr 6 SNP cluster at ~133,850,000 shown in Table 4. The DK center showed strongest association for 2 SNPs at the *CRP* locus, but essentially no apparent association with the top SNPs from the other centers. The AZ center also showed substantial association (p-values from 2.3 x 10^−6^ to 7 x 10^−5^) with SNPs at chr 6, but in loci quite distant from the clusters identified in the overall analysis. Although other SNPs showed association p-values below the genome-wide threshold, there were only 171 participants at the AZ center and there was minimal overlap with loci in the overall analysis or the other centers. Chromosome 11 also contained a large cluster of SNPs with maximum association p-values of ~1 x 10^−6^, but the minor allele frequencies were quite low, leaving the results dependent on as few as 6 individuals from the total of 171.

### Conditional analysis for fine mapping

We conducted linkage analysis conditional on our top SNPs on chromosomes 5 and 6. For model 4, our topmost signal was on chromosome 5 (LOD = 2.00). Our association analysis identified four SNPs on chromosome 5 (p < 7 x 10^−4^), however they were not in the same region as our linkage signal and thus adjusting for the SNPs did not change the linkage signal (LOD = 2.00). For model 3, our best linkage hit was on chromosome 6 (LOD = 1.9). There were two clusters of SNPs on chromosome 6 which were associated with CRP at a significance level of < 7 x 10^−4^. None of these regions overlapped with our linkage signal. The SNPs with at least nominal association with CRP and closest to our linkage region were about 34Mb apart.

On chromosome 1, we identified a 5Mb region from 154,453,788 to 159,730,249 that contained most of the significant SNPs in the literature. Of the 20 SNPs in this region, only rs2794520, rs1205, rs1341665 and rs3091244 were genotyped in our dataset. For the rest of the SNPs we scanned the region plus and minus 500kb to find proxy SNPs. We considered proxy SNPs to be those that are significantly associated with CRP and are in LD (r^2^ ≥ 0.8) with one of the four SNPs in a European population. We found that except two SNPs of the *IL6R* gene and four SNPs (rs1417938, rs4131568, rs1800947, rs3093058) of *CRP*, the remaining 14 SNPs were in strong LD with each other. Since none of the other SNPs in the region were significantly associated with CRP, we conducted a conditional analysis with one variant (rs1205) of the LD block of 14 SNPs. With adjustment for rs1205, the *CRP* SNPs rs2592887, rs1470515, rs2794520 and rs1341665 became non-significant, with p-values of 0.26, 0.35, 0.41 and 0.63 respectively. For chromosome 12, we did not find any SNPs to be in LD with significant SNPs in other populations. However, since our best association signal was on chromosome 12 (*HNF1A)*, we conducted additional analysis conditional on rs2393791 to identify secondary signals. Similar to previous analysis, loci on chromosome 12 were no longer significant and others showed minimal changes. For chromosome 19, we found rs8106922 to be a proxy SNP for rs769450 which was significantly associated with CRP in other populations but not genotyped in our cohort. Analysis conditional on rs8106922, however, showed no change in p-values. In summary, conditional analysis showed loss of nominal association when our initial chr 1 results were adjusted with rs1205 as a covariate; and similar loss of association for chr 12 SNPs in the *HNF1A* region, when adjusting for rs2393791. See S3 Table for details.

### Functional annotation of identified variants

The associated SNPs are grouped into clusters based on their LD patterns (Table 4). We used RegulomeDB [66] and HaploReg [67,68] to functionally annotate *CRP*-associated variants. RegulomeDB showed that rs1969783 and rs1966248 of *TARID* and rs5629931 of *TCF7L2* had a score of 3a (less likely to affect binding) and rs2592887 and rs1205 of *CRP*, rs4895389 of *TARID* and rs3405329 and rs6721844 of *LDAH* had lower scores of 4 (minimal binding evidence). The LDs shown by HaploReg (S4 Table), based on European populations by default, are very similar to the LD patterns found in the present study. In addition, several SNPs overlap with promoter and enhancer histone marks and DNase hypersensitivity regions in various tissues (S4 Table).

## Discussion

The results reported here further inform our understanding of inherited genetic influences on baseline serum CRP levels, by examining a family-based sample with unique ethnic and environmental characteristics, through the use of linkage, focused SNP association analyses, and bio-informatic methods. Previously unrecognized loci suggesting an association with serum CRP levels include a linkage signal at chr 6, 181–194 cM and three SNPs with among the lowest association p-values within our study, located approximately 34 Mb centromeric to the linkage peak. Further support for an effect from this region is seen in center specific linkage and SNP association analysis from Oklahoma. The MetaboChip genotype association results also support earlier findings in proximity to the *CRP* gene (chr 1), the *KCNE4* and *GCKR* genes (chr 2), *HNF1A* (chr 12), and *TOMM40*, *APOE* (chr 19) genes that have been previously associated with *CRP* expression, as noted in Table 1. In addition, the clustering of groups of highly associated SNPs within very limited regions on chromosomes 5, 6, 9, 10, and 17 suggests the existence of novel loci, even though the p-values are above the Bonferroni, genome-wide adjusted threshold. Fine mapping suggested lack of secondary associated SNPs at two regions near the *CRP* and *HNF1A* genes.

The heritability estimate of CRP from the present study is 0.33 (p<1.3 x 10^−20^) compared with similarly-adjusted, previous findings for American Indians (0.38) [32], African Americans (0.45) [69], Chinese (0.38) [70] and non-Hispanic whites (0.40) [57]. The strongest linkage signal, across all centers, was between 189 and 191 cM on chr 6 with adjustment for typical demographic variables; and there was minimal attenuation with further adjustment for DM and hypertensive status. Center specific analyses in both DK and OK revealed linkage peaks in the same chr 6 region, however there was an absence of signal in the AZ center, perhaps due to the small number of participants there. While higher LOD scores were seen in some center-specific analyses, only a LOD of 1.09 in the DK center corresponded with the previously identified chr 19 locus [20,24,71,72], otherwise there seemed to be no correlation between centers, with the MetaboChip association results, or with reports in the literature, as summarized in Table 1. As CRP levels are clearly correlated with measures of obesity [73] and there is a genetic correlation between physical activity and LDL-C [44], it is possible that inclusion of covariates of adiposity, lipids and blood pressure could result in "over-adjustment", reducing power to detect linkage.

While there have been relatively few linkage studies of CRP [74–76], Ding et al reported a LOD of 0.49 on chr 6, 187cM among African Americans [18], and a LOD score of 1.7 (107cM) on chr 10 [18]. While the present linkage results failed to replicate this chr 10 result in American Indians, a suggestive cluster of SNPs was found approximately 30 Mb proximal to this locus. Another region on chr 6 (6q16.1) has been associated with plasma CRP levels in a cohort of Filipino women [25], and lies within ~38 Mb of the present findings at 133,000,000.

Inspecting loci where SNPs influencing CRP expression have been reported, for example, on chr 1 (at the *CRP* gene) [24,77], chromosomes 2 [21,64], 12 [72,77], and 19 [20,24], our results failed to show a linkage signal, with the highest LOD score (1.41) at chr 19, 96cM (*APOE* and *TOMM40* genes).

The MetaboChip data showing 12 SNPs clustered in a span of about 150Kb at position 12,800,000 and another 5 SNPs within 55Kb around 133,850,000 in the 6p22.3 and 6q22.31 regions may represent extended regions of LD which contain a functional variant influencing CRP levels. The first cluster of 12 SNPs is intronic to the *PHACTR1* gene, which plays a role in endothelial cell survival and is associated with susceptibility to myocardial infarction and coronary artery disease [78]. The second cluster of 5 SNPs is within the TCF21 antisense RNA inducing promoter demethylation (*TARID*) gene [79]. Variants within *TARID* or its target (ie *TCF21*) are associated with coronary artery disease [80,81], blood pressure [82], cis-effects on circulating cytokines [83], and visceral fat [84]. Directly between the above two clusters, lies rs7740975 (2.2 X 10^−5^ p-value for association in present study), which is intronic to solute carrier family 35 member F1 (*SLC35F1*), a member of the SLC35 family of transporters which aid in the formation of glycoproteins in the Golgi apparatus and endoplasmic reticulum [85]. Variants of this member of the solute carrier gene family have been associated with a number of cardiovascular disease phenotypes related to hypertension [86], congestive heart failure [87], obesity [88], heart rate [89], and electrocardiographic QT interval [90], as have some polymorphisms of the *CRP* gene [17,91–93]. Of note, the gene G protein-coupled receptor class C group 6 member A (*GPRC6A*) is only 500 Kb distal to this SNP and has demonstrated effects on CRP levels [21]. A search of dbSNP failed to reveal any other significant citations of SNPs from this region on chr 6 in relation to effects on serum levels of CRP.

Further examination of the MetaboChip results in relation to the apparent clusters of associated SNPs, or possible haplotypes, we find that the group on chr 1 between 159,683,149 and 159,721,769 very clearly overlap the *CRP* gene, as well as contain documented, functional SNPs such as the 3' UTR SNP, rs1205 [24,25]. The rs2592887 SNP in this region is in linkage disequilibrium with rs876537, which is associated with CRP in both European and African American populations [94].

The chr 2 cluster between 20,961,892 and 20,989,723 is approximately 25 Kb from the 3' end of the apolipoprotein B (*APOB*) gene. This gene is intricately involved with lipid metabolism and regulation, as well as associated with a number of cardiovascular disease entities [95]. This set of SNPs is also 150 Kb 5' from the lipid droplet associated hydrolase (*LDAH*) gene, similarly involved in lipid metabolism and demonstrating increased expression within the macrophages of human atherosclerotic lesions [96].

Five SNPs within 200 Kb on chr 5 reside very near the *CEPB4* gene, variants of which have been related to control of inflammation and obesity [97]. Within 6 Kb of *SLC2A6* on chr 9, a group of 5 SNPs is nominally associated with CRP. This gene is involved with hexose transport in brain, spleen and leukocytes [98], as well as increasingly expressed in chronic lymphocytic leukemia [99], but a PubMed search reveals no apparent relevance to clinical inflammation or CRP expression.

Another suggestive cluster is found on chr 10, comprising 10 SNPs within 243 Kb, all but one of which are intronic to the transcription factor 7 like 2 (*TCF7L2*) gene. TCF7L2 is instrumental in the Wnt signaling pathway [100] and variants are well known to be associated with risk of DM and its complications [101]. Variants of this gene also alter CRP levels in response to drug treatment [102].

Consistent with other studies, there are 19 SNPs within a 43 Kb span on chr 12 encompassing the *HNF1A* gene, a hepatic transcription factor which has been repeatedly found to affect CRP expression [24,77], as well as *C12orf43*, variants of which have been linked to cardiovascular disease [103] and CRP expression [20]. Mutations of HNF1A are known to cause maturity DM of youth, type 3 (MODY3) [104], and polymorphisms are associated with risk of DM and atherosclerotic vascular disease [105].

Lastly, 3 SNPs on chr 17 lie within 17 Kb, within or between *MYL4* and *CDC27*, the latter known to influence TGF-beta [106], a strong modulator of inflammatory response [107].

A rather extensive literature exists associating SNPs with serum CRP [20,21,24,25,72,77,108]. Some of the more compelling reports are summarized in Table 1. Besides *CRP*, the *LEPR* region on chr 1, has suggestive findings in the current study; and three SNPs intronic to *JAK1* show nominal significance. The latter gene plays a key role in immune response pathways [109] and is within 400 Kb of *LEPR*. Conversely, the *IL6R* region fails to indicate any signal, including rs4129267 (p = 0.17). Our results in the *GCKR* region reveal a cluster of SNPs, with maximal association p-values of 3.5 X 10^−3^. Although 31 SNPs were genotyped in the *EPHA* and many in the *IL6* regions, no indications of association with CRP were found. Current findings related to the *HNF1A* gene are noted above.

Our results highlight 5 SNPs in the *APOE*, *TOMM40*, *APOC1* area, with p-values for association all less than 6.8 X 10^−3^. The *TOMM40* protein is a component of the mitochondrial membrane and mutations appear to contribute to risk of Alzheimer's disease and other aging phenomenon [110,111]. The *APOE* and *APOC1* genes play important roles in lipid metabolism and are associated with clinical conditions dependent on this function [112,113].

Like most genetic association studies, we identified several noncoding variants associated with CRP levels. Although noncoding regions do not affect mRNA sequence, they may regulate other factors involved in the transcription or regulation of the genes. We used RegulomeDB [66] and HaploReg [67,68] to functionally annotate variants; and several, such as rs1205, rs35131127, rs1969783, and rs1169310 showed potential evidence of functionality. (S4 Table)

Limitations of this study include marginally significant findings after conservative, Bonferroni adjustment for multiple testing in a study population of a moderate sample size. This is ameliorated to some extent by the correlation between many groups of SNPs in the array and the fact that all of the MetaboChip SNPs were chosen for *a priori* evidence of association with cardiovascular phenotypes, which also relate to CRP. The somewhat indirect correspondence between the linkage and SNP association analyses shows different strengths of the methodologies, in that linkage appears more successful at identifying rare and family-specific variants whereas association analysis tends to rely more on common variants, as illustrated in an analysis by He et al [114]. Differences may also arise from the fact that the MetaboChip cohort excluded those with DM, perhaps minimizing the effect of variants that both predisposed to diabetes/obesity and increased CRP level. Our replication of an interaction (gender by microarray determined genotype) at certain loci (eg at rs12723357 on chr 1 and at rs17301021 on chr 15) is an important reminder that the potential for systematic, microarray genotyping errors is a problem that warrants careful attention [52]. An additional concern involved identified clusters of SNPs within constricted regions that showed strong association with CRP; but also showed uniformly marginal HWE p-values (eg 9 SNPs on chr 6), all within 250 Kb and none of which with HWE p-values greater than 2.2 X 10^−4^. In contrast to the clusters we thought pointed to regions harboring a functional variant (with HWE results well within an expected distribution), the anomalous clusters were interpreted as due to haplotypes identifying unique center background and thus spuriously associated with CRP due to the recognized differences in CRP by center. The underlying difference in CRP between centers could be due to genetic influences; but could also reflect environmental factors as well. In either case, this probably represents an example of population stratification, when the analysis addresses all centers combined.

The strengths of this study include a population-based ascertainment of samples from communities with unique environmental and genetic backgrounds, extensive covariate information collected in a prospective manner, and the use of two complementary genetic analysis methods. From a broader perspective, the SHS [44] has represented a relatively successful collaboration with the participating tribal communities since inception in 1998. Sustaining a mutually beneficial engagement at this level requires considerable effort from both parties; but we feel the history of the Strong Heart Study can provide a useful model for this type of research.

## Supporting information

S1 Table Genotype by gender interaction for two anomalous SNPs. (DOCX)

S2 Table SNP clusters of interest. (DOCX)

S3 Table Association analysis of SNPs conditional on rs1205 and rs2393791 genotype. (DOCX)

S4 Table Bioinformatic analysis of SNP clusters associated with serum CRP levels utilizing HaploReg and RegulomeDB. (DOCX)

S1 Fig Locus zoom (A) and linkage disequilibrium plots (B) for chromosome 1.
(PDF)

S2 Fig Locus zoom (A) and linkage disequilibrium plots (B) for chromosome 2.
(PDF)

S3 Fig Locus zoom (A) and linkage disequilibrium plots (B) for chromosome 5.
(PDF)

S4 Fig Locus zoom (A) and linkage disequilibrium plots (B) for chromosome 6a.
(PDF)

S5 Fig Locus zoom (A) and linkage disequilibrium plots (B) for chromosome 6b.
(PDF)

S6 Fig Locus zoom (A) and linkage disequilibrium plots (B) for chromosome 9.
(PDF)

S7 Fig Locus zoom (A) and linkage disequilibrium plots (B) for chromosome 10.
(PDF)

S8 Fig Locus zoom (A) and linkage disequilibrium plots (B) for chromosome 12.
(PDF)

S9 Fig Locus zoom (A) and linkage disequilibrium plots (B) for chromosome 17.
(PDF)

S10 Fig Locus zoom (A) and linkage disequilibrium plots (B) for chromosome 19.
(PDF)
