# Supplementary material for: Genetic analysis of hsCRP in American Indians: The Strong Heart Family Study
Source: PLoS One. 2019 Oct 17;14(10):e0223574. doi: 10.1371/journal.pone.0223574 (PMC6797125; doi:10.1371/journal.pone.0223574)
Supplement: S1 Table — (DOCX) [file pone.0223574.s001.docx]

Supplementary Table S1: Genotype by gender interaction for two anomalous SNPs.

| **rs12723357** | | | |
| --- | --- | --- | --- |
|  | Genotype | |  |
|  | A/A | G/A |  |
| Female | 1355 | 4 |  |
| Male | 5 | 902 | P<0.001 |
| **rs12734338** | | | |
|  | A/A | G/A |  |
| Female | 1355 | 4 |  |
| Male | 5 | 902 | P<0.001 |
