# Supplementary material for: Genetic analysis of hsCRP in American Indians: The Strong Heart Family Study
Source: PLoS One. 2019 Oct 17;14(10):e0223574. doi: 10.1371/journal.pone.0223574 (PMC6797125; doi:10.1371/journal.pone.0223574)
Supplement: S2 Table — (DOCX) [file pone.0223574.s002.docx]

Supplementary Table S2: SNP clusters of interest.

| **SNP clusters of interest** | | | | | | | |
| --- | --- | --- | --- | --- | --- | --- | --- |
| **SNP** | **Gene*** | **Chr:** | **Min/maj**  **Allele**** | **z-score***** | **P** | **Physical**  **Coordinates****** | **MAF** |
| rs2592887 | *CRP* | 1 | A/G | -3.59 | 3.4 X 10^-4^ | 159,683,149 | .477 |
| rs1470515 | *CRP* | 1 | A/G | -3.55 | 3.8 X 10^-4^ | 159,683,809 | .483 |
| rs2794520 | *CRP* | 1 | A/G | -3.36 | 7.8 X 10^-4^ | 159,709,026 | .495 |
| rs1205 | *CRP* | 1 | A/G | -3.43 | 6.0 X 10^-4^ | 159,712,443 | .495 |
| rs1341665 | *CRP* | 1 | A/G | -3.50 | 4.7 X 10^-4^ | 159,721,769 | .485 |
| rs2337382 | *LDAH / APOB* | 2 | A/G | 3.81 | 1.4 X 10^-4^ | 20,961,892 | .374 |
| rs6706783 | *LDAH / APOB* | 2 | A/T | -3.74 | 1.9 X 10^-4^ | 20,968,544 | .376 |
| rs35131127 | *LDAH / APOB* | 2 | A/G | -3.84 | 1.2 X 10^-4^ | 20,983,066 | .368 |
| rs56327713 | *LDAH / APOB* | 2 | A/C | 3.85 | 1.2 X 10^-4^ | 20,983,988 | .368 |
| rs34059329 | *LDAH / APOB* | 2 | A/G | 3.80 | 1.5 X 10^-4^ | 20,986,919 | .366 |
| rs6721844 | *LDAH / APOB* | 2 | A/C | 3.63 | 2.9 X 10^-4^ | 20,989,723 | .372 |
| rs360017 | *BOD1* | 5 | A/G | -3.48 | 5.0 X 10^-4^ | 173,780,350 | .073 |
| rs17076582 | *CPEB4* | 5 | A/G | -3.93 | 8.5 X 10^-5^ | 173,839,020 | .078 |
| rs56018556 | *CPEB4* | 5 | A/G | -3.75 | 1.8 X 10^-4^ | 173,839,631 | .091 |
| rs59335160 | *CPEB4* | 5 | A/G | -3.69 | 2.2 X 10^-4^ | 173,840,260 | .077 |
| rs1994236 | *C5orf47* | 5 | A/G | -3.76 | 1.7 X 10^-4^ | 173,974,340 | .182 |
| rs475543 | *PHACTR1* | 6 | A/G | -3.49 | 4.9 X 10^-4^ | 12,717,924 | .367 |
| rs560810 | *PHACTR1* | 6 | A/C | 3.49 | 4.9 X 10^-4^ | 12,722,755 | .367 |
| rs581046 | *PHACTR1* | 6 | A/G | 3.38 | 7.2 X 10^-4^ | 12,725,709 | .367 |
| rs2026457 | *PHACTR1* | 6 | A/G | -3.92 | 8.7 X 10^-5^ | 12,825,573 | .389 |
| rs2026458 | *PHACTR1* | 6 | A/G | 3.71 | 2.1 X 10^-4^ | 12,825,642 | .436 |
| rs9349344 | *PHACTR1* | 6 | A/G | 3.90 | 9.8 X 10^-5^ | 12,826,247 | .389 |
| rs9349346 | *PHACTR1* | 6 | A/T | 3.92 | 8.8 X 10^-5^ | 12,827,102 | .389 |
| rs9395172 | *PHACTR1* | 6 | A/G | -3.89 | 1.0 X 10^-4^ | 12,850,302 | .384 |
| rs6917097 | *PHACTR1* | 6 | A/G | 3.98 | 6.9 X 10^-5^ | 12,851,536 | .383 |
| rs4714930 | *PHACTR1* | 6 | A/C | 3.71 | 2.1 X 10^-4^ | 12,852,246 | .380 |
| rs9472752 | *PHACTR1* | 6 | A/G | 4.01 | 6.1 X 10^-5^ | 12,863,902 | .383 |
| rs9296495 | *PHACTR1* | 6 | A/G | -4.01 | 6.1 X 10^-5^ | 12,865,417 | .383 |
| rs4895389 | *TARID* | 6 | A/G | 3.99 | 6.7 X 10^-5^ | 133,838,014 | .348 |
| rs1969783 | *TARID* | 6 | A/G | 4.04 | 5.3 X 10^-5^ | 133,838,261 | .350 |
| rs1966248 | *TARID* | 6 | A/G | -4.07 | 4.8 X 10^-5^ | 133,838,484 | .342 |
| rs2327429 | *TARID* | 6 | A/G | -3.47 | 5.3 X 10^-4^ | 133,888,699 | .470 |
| rs12190287 | *TCF21 / TARID* | 6 | C/G | 3.70 | 2.2 X 10^-4^ | 133,893,387 | .492 |
| rs28453139 | *SLC2A6* | 9 | A/G | -3.79 | 1.5 X 10^-4^ | 133,485,870 | .080 |
| rs28718919 | *SLC2A6* | 9 | A/C | 3.73 | 1.9 X 10^-4^ | 133,486,512 | .081 |
| rs28360841 | *SLC2A6* | 9 | A/G | -3.65 | 2.6 X 10^-4^ | 133,489,879 | .084 |
| rs28678509 | *SLC2A6* | 9 | A/G | -3.39 | 7.1 X 10^-4^ | 133,492,555 | .081 |
| rs28536509 | *SLC2A6* | 9 | A/G | -3.44 | 5.7 X 10^-4^ | 133,495,031 | .079 |
| rs4267006 | *TCF7L2* | 10 | A/C | -3.55 | 3.8 X 10^-4^ | 112,999,020 | .068 |
| rs4575195 | *TCF7L2* | 10 | A/C | -4.02 | 5.9 X 10^-5^ | 113,005,988 | .108 |
| rs4132670 | *TCF7L2* | 10 | A/G | -4.06 | 5.0 X 10^-5^ | 113,008,012 | .108 |
| rs55899248 | *TCF7L2* | 10 | A/G | 3.78 | 1.6 X 10^-4^ | 113,013,849 | .069 |
| rs55853916 | *TCF7L2* | 10 | A/G | -3.78 | 1.6 X 10^-4^ | 113,022,191 | .069 |
| rs55972445 | *TCF7L2* | 10 | A/C | -3.78 | 1.6 X 10^-4^ | 113,023,031 | .069 |
| rs56299331 | *TCF7L2* | 10 | A/G | -3.63 | 2.8 X 10^-4^ | 113,028,677 | .059 |
| rs72826094 | *TCF7L2* | 10 | A/T | 3.62 | 3.0 X 10^-4^ | 113,041,729 | .059 |
| rs61872786 | *TCF7L2* | 10 | A/G | -3.58 | 3.4 X 10^-4^ | 113,046,938 | .061 |
| rs11819509 | *TCF7L2* | 10 | A/G | -3.40 | 6.8 X 10^-4^ | 113,241,955 | .020 |
| rs7953249 | *HNF1A* | 12 | a/G | 3.54 | 4.0 X 10^-4^ | 120,965,921 | .426 |
| rs1169288 | *HNF1A* | 12 | A/C | 3.54 | 4.0 X 10^-4^ | 120,978,847 | .414 |
| rs2244608 | *HNF1A* | 12 | a/G | 3.85 | 3.9 X 10^-4^ | 120,979,185 | .386 |
| rs7979473 | *HNF1A* | 12 | a/G | -4.44 | 9.0 X 10^-6^ | 120,982,457 | .413 |
| rs1183910 | *HNF1A* | 12 | a/G | -3.86 | 1.1 X 10^-4^ | 120,983,004 | .382 |
| rs2393791 | *HNF1A* | 12 | a/G | 4.59 | 4.5 X 10^-6^ | 120,986,153 | .409 |
| rs7310409 | *HNF1A* | 12 | a/G | -4.57 | 4.8 X 10^-6^ | 120,987,058 | .408 |
| rs35379941 | *HNF1A* | 12 | a/T | 3.96 | 7.6 X 10^-5^ | 120,987,586 | .115 |
| rs2264782 | *HNF1A* | 12 | a/G | -3.41 | 6.6 X 10^-4^ | 120,994,800 | .441 |
| rs2259852 | *HNF1A* | 12 | a/G | -3.41 | 6.4 X 10^-4^ | 120,997,030 | .441 |
| rs2259816 | *HNF1A* | 12 | A/C | -3.36 | 7.7 X 10^-4^ | 120,997,784 | .442 |
| rs1169306 | *HNF1A* | 12 | a/G | -3.36 | 7.9 X 10^-4^ | 121,000,508 | .442 |
| rs735396 | *HNF1A* | 12 | a/G | 3.36 | 7.8 X 10^-4^ | 121,001,041 | .442 |
| rs1169309 | *HNF1A* | 12 | A/C | -3.36 | 7.7 X 10^-4^ | 121,001,389 | .442 |
| rs1169310 | *HNF1A* | 12 | a/G | -3.36 | 7.8 X 10^-4^ | 121,001,630 | .442 |
| rs1169312 | *C12orf43* | 12 | A/C | -3.40 | 6.8 X 10^-4^ | 121,003,658 | .443 |
| rs1169313 | *C12orf43* | 12 | a/G | 3.46 | 5.5 X 10^-4^ | 121,004,867 | .443 |
| rs191722051 | *C12orf43* | 12 | a/G | 3.43 | 6.0 X 10^-4^ | 121,006,640 | .444 |
| rs2257962 | *C12orf43* | 12 | a/G | 3.40 | 6.8 X 10^-4^ | 121,008,005 | .443 |
| rs858671 | *CDC27* | 17 | A/C | -3.68 | 2.3 X 10^-4^ | 47,194,314 | .226 |
| rs9894365 | *MYL4* | 17 | a/G | 3.81 | 1.4 X 10^-4^ | 47,207,421 | .226 |
| rs11079763 | *MYL4* | 17 | A/C | -3.70 | 2.2 X 10^-4^ | 47,211,423 | .223 |
| rs2075650 | *TOMM40* | 19 | a/G | 3.23 | 1.2 X 10^-3^ | 44,892,362 | 0.15 |
| rs8106922 | *TOMM40* | 19 | a/G | -2.70 | 6.8 X 10^-3^ | 44,898,409 | 0.31 |
| rs7259620 | *TOMM40* | 19 | A/C/G | 2.84 | 4.5 X 10^-3^ | 44,904,531 | 0.42 |
| rs769449 | *APOE* | 19 | a/G | -3.34 | 8.4 X 10^-4^ | 44,906,745 | 0.13 |
| rs769450 | *APOE* | 19 | a/G | 1.99 | 4.7 X 10^-2^ | 44,907,187 | 0.15 |
| rs56131196 | *APOC1* | 19 | a/G | -3.28 | 1.0 X 10^-3^ | 44,919,589 | 0.09 |

* Most proximal candidate gene

** Minor allele is effect allele, major is referent

*** inverse weighted average of three centers

**** GRCh38.p7, dbSNP build 150
