# Supplementary material for: Genetic analysis of hsCRP in American Indians: The Strong Heart Family Study
Source: PLoS One. 2019 Oct 17;14(10):e0223574. doi: 10.1371/journal.pone.0223574 (PMC6797125; doi:10.1371/journal.pone.0223574)
Supplement: S3 Table — (DOCX) [file pone.0223574.s003.docx]

Supplementary Table S3: Association analysis of SNPs conditional on rs1205 and rs2393791 genotype.

| **SNP clusters of interest** | | | | | | |
| --- | --- | --- | --- | --- | --- | --- |
|  |  | **Standard** | **Conditional analysis (rs1205)** | | **Conditional analysis (rs2393791)** | |
|  |  | **Pvalue** | **Pvalue** | **β (SE)** | **Pvalue** | **β (SE)** |
| rs2592887 | *CRP* | 3.4 X 10^-4^ | 0.26 | 0.09 (0.08) | 3 x 10^-3^ | -0.11 (0.03) |
| rs1470515 | *CRP* | 3.8 X 10^-4^ | 0.35 | 0.08 (0.08) | 3 x 10^-3^ | -0.11 (0.03) |
| rs2794520 | *CRP* | 7.8 X 10^-4^ | 0.41 | -0.65 (0.78) | 4 x 10^-3^ | -0.10 (0.04) |
| rs1341665 | *CRP* | 4.7 X 10^-4^ | 0.63 | 0.07 (0.15) | 4 x 10^-3^ | 0.10 (0.04) |
| rs2337382 | *LDAH / APOB* | 1.4 X 10^-4^ | 1.4 x 10^-4^ | 0.14 (0.04) | 6.8 x 10^-4^ | 0.13 (0.04) |
| rs6706783 | *LDAH / APOB* | 1.9 X 10^-4^ | 1.7 x 10^-4^ | 0.14 (0.04) | 8.6 x 10^-4^ | 0.12 (0.04) |
| rs35131127 | *LDAH / APOB* | 1.2 X 10^-4^ | 1.3 x 10^-4^ | 0.14 (0.04) | 5.7 x 10^-4^ | 0.13 (0.04) |
| rs56327713 | *LDAH / APOB* | 1.2 X 10^-4^ | 1.1 x 10^-4^ | 0.14 (0.04) | 6.0 x 10^-4^ | 0.13 (0.04) |
| rs34059329 | *LDAH / APOB* | 1.5 X 10^-4^ | 1.4 x 10^-4^ | 0.14 (0.04) | 6.0 x 10^-4^ | 0.13 (0.04) |
| rs6721844 | *LDAH / APOB* | 2.9 X 10^-4^ | 2.5 x 10^-4^ | 0.14 (0.04) | 1.0 x 10^-3^ | 0.12 (0.04) |
| rs360017 | *BOD1* | 5.0 X 10^-4^ | 6.6 x 10^-5^ | -0.26 (0.06) | 6.2 x 10^-5^ | -0.26 (0.06) |
| rs17076582 | *CPEB4* | 8.5 X 10^-5^ | 1.8 x 10^-6^ | -0.31 (0.06) | 2.4 x 10^-6^ | -0.30 (0.07) |
| rs56018556 | *CPEB4* | 1.8 X 10^-4^ | 1.7 x 10^-6^ | -0.29 (0.06) | 3.4 x 10^-6^ | -0.28 (0.06) |
| rs59335160 | *CPEB4* | 2.2 X 10^-4^ | 4.7 X 10^-6^ | -0.30 (0.07) | 6.9 x 10^-6^ | -0.29 (0.07) |
| rs475543 | *PHACTR1* | 4.9 X 10^-4^ | 2.4 x 10^-4^ | -0.13 (0.04) | 2.6 x 10^-4^ | -0.13 (0.04) |
| rs560810 | *PHACTR1* | 4.9 X 10^-4^ | 2.4 x 10^-4^ | -0.13 (0.04) | 2.6 x 10^-4^ | -0.13 (0.04) |
| rs581046 | *PHACTR1* | 7.2 X 10^-4^ | 3.8 x 10^-4^ | -0.13 (0.04) | 4.1 x10^-4^ | -0.13 (0.04) |
| rs2026457 | *PHACTR1* | 8.7 X 10^-5^ | 4.6 x 10^-5^ | -0.14 (0.04) | 4.4 x 10^-4^ | -0.14 (0.04) |
| rs2026458 | *PHACTR1* | 2.1 X 10^-4^ | 2.2 x 10^-4^ | 0.13 (0.04) | 3.3 x 10^-4^ | 0.13 (0.04) |
| rs9349344 | *PHACTR1* | 9.8 X 10^-5^ | 5.3 x 10^-5^ | -0.14 (0.04) | 5.1 x 10^-5^ | -0.14 (0.04) |
| rs9349346 | *PHACTR1* | 8.8 X 10^-5^ | 4.5 x10^-5^ | -0.14 (0.04) | 4.4 x 10^-4^ | -0.13 (0.04) |
| rs9395172 | *PHACTR1* | 1.0 X 10^-4^ | 6.8 x 10^-5^ | -0.14 (0.04) | 7.2 x 10^-5^ | -0.14 (0.04) |
| rs6917097 | *PHACTR1* | 6.9 X 10^-5^ | 4.5 x10^-5^ | -0.14 (0.04) | 4.5 x 10^-5^ | -0.14 (0.04) |
| rs4714930 | *PHACTR1* | 2.1 X 10^-4^ | 1.2 x 10^-4^ | -0.14 (0.04) | 1.2 x 10^-4^ | -0.14 (0.04) |
| rs9472752 | *PHACTR1* | 6.1 X 10^-5^ | 4.6 x 10^-5^ | -0.14 (0.04) | 4.8 x 10^-5^ | -0.14 (0.04) |
| rs9296495 | *PHACTR1* | 6.1 X 10^-5^ | 4.6 x 10^-5^ | -0.14 (0.04) | 4.8 x 10^-5^ | -0.14 (0.04) |
| rs4895389 | *TARID* | 6.7 X 10^-5^ | 2.4 x 10^-6^ | -0.17 (0.04) | 5.3 x 10^-6^ | -0.16 (0.04) |
| rs1969783 | *TARID* | 5.3 X 10^-5^ | 1.9 x 10^-6^ | -0.17 (0.04) | 3.7 x 10^-6^ | -0.17 (0.04) |
| rs1966248 | *TARID* | 4.8 X 10^-5^ | 1.5 x 10^-6^ | -0.17 (0.04) | 2.9 x 10^-6^ | -0.17 (.04) |
| rs2327429 | *TARID* | 5.3 X 10^-4^ | 2.2 x 10^-4^ | 0.13 (0.04) | 3.5 x 10^-4^ | 0.13 (0.04) |
| rs12190287 | *TCF21 / TARID* | 2.2 X 10^-4^ | 9.8 x 10^-5^ | 0.14 (0.04) | 1.6 x 10^-4^ | 0.13 (0.04) |
| rs28453139 | *SLC2A6* | 1.5 X 10^-4^ | 6.5 x 10^-5^ | -0.27 (0.07) | 9.2 x 10^-5^ | -0.27 (0.07) |
| rs28718919 | *SLC2A6* | 1.9 X 10^-4^ | 7.1 x 10^-5^ | -0.27 (0.07) | 1.3 x 10^-4^ | -0.26 (0.07) |
| rs28360841 | *SLC2A6* | 2.6 X 10^-4^ | 8.3 x 10^-5^ | -0.26 (0.07) | 1.0 x 10^-4^ | -0.26 (0.07) |
| rs28678509 | *SLC2A6* | 7.1 X 10^-4^ | 3.3 x 10^-4^ | -0.24 (0.07) | 5.0 x 10^-4^ | - 0.24 (0.07) |
| rs28536509 | *SLC2A6* | 5.7 X 10^-4^ | 2.9 x 10^-4^ | -0.25 (0.07) | 3.7 x 10^-4^ | -0.25 (0.07) |
| rs4267006 | *TCF7L2* | 3.8 X 10^-4^ | 5.9 x 10^-4^ | -0.23 (0.07) | 2.2 x 10^-4^ | -0.25 (0.07) |
| rs4575195 | *TCF7L2* | 5.9 X 10^-5^ | 6.6 x 10^-5^ | -0.22 (0.06) | 2.4 x 10^-5^ | -0.24 (0.06) |
| rs4132670 | *TCF7L2* | 5.0 X 10^-5^ | 5.9 x 10^-5^ | -0.22 (0.06) | 2.1 x 10^-5^ | -0.24 (0.06) |
| rs55899248 | *TCF7L2* | 1.6 X 10^-4^ | 1.4 x 10^-4^ | -0.25 (0.07) | 5.7 x 10^-5^ | -0.27 (0.07) |
| rs55853916 | *TCF7L2* | 1.6 X 10^-4^ | 1.4 x 10^-4^ | -0.25 (0.07) | 5.7 x 10^-5^ | -0.27 (0.07) |
| rs55972445 | *TCF7L2* | 1.6 X 10^-4^ | 1.4 x 10^-4^ | -0.25 (0.07) | 5.7 x 10^-5^ | -0.27 (0.07) |
| rs56299331 | *TCF7L2* | 2.8 X 10^-4^ | 2.3 x 10^-4^ | -0.26 (0.07) | 1.0 x 10^-4^ | -0.28 (0.07) |
| rs72826094 | *TCF7L2* | 3.0 X 10^-4^ | 2.4 x 10^-4^ | -0.26 (0.07) | 1.0 x 10^-4^ | -0.28 (0.07) |
| rs61872786 | *TCF7L2* | 3.4 X 10^-4^ | 2.2 X 10^-4^ | -0.26 (0.07) | 8.9 x 10^-5^ | -0.28 (0.07) |
| rs11819509 | *TCF7L2* | 6.8 X 10^-4^ | 1.1 x 10^-3^ | -0.45 (0.14) | 1.6 x 10^-3^ | -0.44 (0.14) |
| rs7953249 | *HNF1A* | 4.0 X 10^-4^ | 2.4 x 10^-4^ | -0.13 (0.04) | 0.41 | 0.03 (0.04) |
| rs1169288 | *HNF1A* | 4.0 X 10^-4^ | 6.7 x 10^-4^ | -0.12 (0.04) | 0.45 | 0.03 (0.04) |
| rs2244608 | *HNF1A* | 3.9 X 10^-4^ | 1.3 x10^-4^ | -0.14 (0.04) | 0.56 | 0.02 (0.04) |
| rs7979473 | *HNF1A* | 9.0 X 10^-6^ | 6.3 x 10^-6^ | -0.16 (0.04) | 0.74 | 0.01 (0.04) |
| rs1183910 | *HNF1A* | 1.1 X 10^-4^ | 1.3 x 10^-4^ | -0.14 (0.04) | 0.49 | 0.03 (0.04) |
| rs7310409 | *HNF1A* | 4.8 X 10^-6^ | 3.6 x 10^-6^ | -0.17 (0.04) | 0.82 | 0.01 (0.04) |
| rs35379941 | *HNF1A* | 7.6 X 10^-5^ | 1.0 x 10^-4^ | 0.20 (0.05) | 2.5 x 10^-2^ | 0.12 (0.05) |
| rs2264782 | *HNF1A* | 6.6 X 10^-4^ | 1.9 x10^-3^ | -0.11 (0.04) | 0.62 | 0.02 (0.04) |
| rs2259852 | *HNF1A* | 6.4 X 10^-4^ | 2.2 x 10^-3^ | -0.11 (0.04) | 0.62 | 0.02 (0.04) |
| rs2259816 | *HNF1A* | 7.7 X 10^-4^ | 2.3 x 10^-3^ | -0.11 (0.04) | 0.58 | 0.02 (0.04) |
| rs1169306 | *HNF1A* | 7.9 X 10^-4^ | 2.4 x 10^-3^ | -0.11 (0.04) | 0.58 | 0.02 (0.04) |
| rs735396 | *HNF1A* | 7.8 X 10^-4^ | 2.4 x 10^-3^ | -0.11 (0.04) | 0.58 | 0.02 (0.04) |
| rs1169309 | *HNF1A* | 7.7 X 10^-4^ | 2.4 x 10^-3^ | -0.11 (0.04) | 0.58 | 0.02 (0.04) |
| rs1169310 | *HNF1A* | 7.8 X 10^-4^ | 2.4 x 10^-3^ | -0.11 (0.04) | 0.58 | 0.02 (0.04) |
| rs1169312 | *C12orf43* | 6.8 X 10^-4^ | 2.0 x 10^-3^ | -0.11 (0.04) | 0.61 | 0.02 (0.04) |
| rs1169313 | *C12orf43* | 5.5 X 10^-4^ | 2.0 x 10^-3^ | -0.11 (0.04) | 0.61 | 0.02 (0.04) |
| rs191722051 | *C12orf43* | 6.0 X 10^-4^ | 2.0 x 10^-3^ | -0.11 (0.04) | 0.61 | 0.02 (0.04) |
| rs2257962 | *C12orf43* | 6.8 X 10^-4^ | 2.0 x 10^-3^ | -0.11 (0.04) | 0.61 | 0.02 (0.04) |
| rs858671 | *CDC27* | 2.3 X 10^-4^ | 5.8 x 10^-5^ | -0.17 (0.04) | 9.5 x 10^-5^ | -0.16 (0.04) |
| rs9894365 | *MYL4* | 1.4 X 10^-4^ | 3.7 x 10^-5^ | -0.17 (0.04) | 6.5 x 10^-5^ | -0.17 (0.04) |
| rs11079763 | *MYL4* | 2.2 X 10^-4^ | 4.8 x 10^-5^ | -0.17 (0.04) | 8.0 x 10^-5^ | -0.17 (0.04) |
| rs2075650 | *TOMM40* | 1.2 X 10^-3^ | 1.3 x 10^-2^ | -0.12 (0.05) | 1.9 x 10^-2^ | -0.12 (0.05) |
| rs8106922 | *TOMM40* | 6.8 X 10^-3^ | 1.3 x 10^-2^ | 0.10 (0.04) | 1.5 x 10^-2^ | 0.10 (0.04) |
| rs7259620 | *TOMM40* | 4.5 X 10^-3^ | 0.005 | 0.10 (0.04) | 1.1 x 10^-2^ | 0.09 (0.04) |
| rs769449 | *APOE* | 8.4 X 10^-4^ | 0.008 | -0.14 (0.05) | 1.3 x 10^-2^ | -0.13 (0.05) |
