# Supplementary material for: Genetic analysis of hsCRP in American Indians: The Strong Heart Family Study
Source: PLoS One. 2019 Oct 17;14(10):e0223574. doi: 10.1371/journal.pone.0223574 (PMC6797125; doi:10.1371/journal.pone.0223574)
Supplement: S4 Table — (DOCX) [file pone.0223574.s004.docx]

Supplementary Table S4: Bioinformatic analysis of SNP clusters associated with serum CRP levels utilizing HaploReg [58] and RegulomeDB [57]

| SNP | Gene | LD | AI**  current study | AFR | AMR | ASN | EUR | Promoter histone marks | Enhancer histone marks | DNAse | Proteins bound | Motifs changed |
| --- | --- | --- | --- | --- | --- | --- | --- | --- | --- | --- | --- | --- |
|  |  |  | Minor allele frequency | | | | |  |  |  |  |  |
| rs2592887* | *CRP* | 1 | 0.48 | 0.52 | 0.42 | 0.59 | 0.39 |  |  |  |  | 6 altered motifs |
| rs1470515 |  | 0.81 | 0.48 | 0.22 | 0.39 | 0.59 | 0.36 |  |  |  |  |  |
| rs2794520 |  | 0.99 | 0.50 | 0.20 | 0.36 | 0.60 | 0.31 |  |  |  |  | COMP1,GR, HNF4 |
| rs1205* |  | 1 | 0.50 | 0.18 | 0.36 | 0.60 | 0.31 | LIV | GI, PANC, LIV | LNG |  | GR |
| rs1341665 |  | 1 | 0.49 | 0.20 | 0.37 | 0.63 | 0.32 |  |  |  |  | 5 altered motifs |
| rs2337382* | *LDAH / APOB* | 1 | 0.37 | 0.54 | 0.58 | 0.89 | 0.59 |  | 5 tissues |  |  |  |
| rs6706783 |  | 0.93 | 0.38 | 0.52 | 0.57 | 0.89 | 0.58 |  | IPSC |  |  |  |
| rs35131127 |  | 0.89 | 0.37 | 0.87 | 0.60 | 0.89 | 0.58 |  | ESDR, KID, MUS | KID, MUS |  |  |
| rs56327713 |  | 0.89 | 0.37 | 0.88 | 0.60 | 0.89 | 0.58 |  | 10 tissues | ESDR,IPSC |  | 5 altered motifs |
| rs34059329 |  | 0.88 | 0.37 | 0.89 | 0.60 | 0.89 | 0.58 | FAT, MUS | 4 tissues |  | CEBPB | ERalpha-a |
| rs6721844 |  | 0.85 | 0.37 | 0.88 | 0.59 | 0.89 | 0.58 |  | ESDR,FAT MUS |  |  |  |
| rs13214585* | *FRK* | 1 | 0.08 | 0.03 | 0.23 | 0.01 | 0.23 |  | ESC |  |  | AIRE,Mef2,RXRA |
| rs1933738* |  | 1 | 0.07 | 0.02 | 0.22 | 0.01 | 0.19 |  |  |  |  | NRSF,SP2 |
| rs12193094* | *COL10A1* | 1 | 0.08 | 0.02 | 0.23 | 0.00 | 0.27 |  | 7 tissues |  |  | 4 altered motifs |
| rs7740975 | *SLC35F1* | 1 | 0.11 | 0.16 | 0.24 | 0.06 | 0.37 |  |  |  |  | Foxp1,PLZF |
| rs4895389* | *TARID* | 1 | 0.35 | 0.36 | 0.52 | 0.47 | 0.33 | 21 tissues | BRST, BLD | 31 tissues | 10 bound proteins |  |
| rs1969783 |  | 0.9 | 0.35 | 0.36 | 0.51 | 0.47 | 0.30 | 20 tissues | BRST, BLD, HRT | 20 tissues | POL2  CTCF  HAE2F1 |  |
| rs1966248 |  | 0.9 | 0.35 | 0.66 | 0.54 | 0.48 | 0.30 | IPSC, BLD | IPSC, PLCNT PANC |  |  | 7 altered motifs |
| rs4267006* | *TCF7L2* | 1 | 0.07 | 0.01 | 0.17 | 0.00 | 0.25 |  | 5 tissues |  |  | 8 altered motifs |
| rs55899248 |  | 0.93 | 0.07 | 0.04 | 0.17 | 0.00 | 0.24 |  | LNG, LIV |  |  | TCF12 |
| rs55853916 |  | 0.95 | 0.07 | 0.02 | 0.17 | 0.00 | 0.24 |  | 18 tissues | 6 tissues |  | 10 altered motifs |
| rs55972445 |  | 0.95 | 0.07 | 0.03 | 0.17 | 0.00 | 0.24 |  | 10 tissues | HRT, MUS |  | Egr-1,Ets,Znf143 |
| rs56299331* |  | 1 | 0.06 | 0.03 | 0.17 | 0.00 | 0.21 | 6 tissues | 18 tissues | 9 tissues |  |  |
| rs72826094 |  | 0.99 | 0.06 | 0.02 | 0.17 | 0.00 | 0.21 |  | 14 tissues |  |  | 6 altered motifs |
| rs61872786 |  | 0.94 | 0.06 | 0.04 | 0.17 | 0.00 | 0.21 |  | 13 tissues | LNG |  | 12 altered motifs |
| rs2264782* | *HNF1A* | 1 | 0.44 | 0.14 | 0.41 | 0.52 | 0.37 |  |  | LNG | SP1, ZNF263 | Pbx-1 |
| rs1169306 |  | 0.98 | 0.44 | 0.14 | 0.42 | 0.52 | 0.37 |  | 6 tissues | PLCNT, THYM |  |  |
| rs1169309 |  | 0.98 | 0.44 | 0.14 | 0.42 | 0.52 | 0.37 |  | 8 tissues | THYM, LIV |  | Mtf-1 |
| rs1169310 |  | 0.98 | 0.44 | 0.14 | 0.42 | 0.52 | 0.37 |  | 7 tissues | 6 tissues | POL2 | ER alpha-a, Esr2, RXRA |
| rs2259852 |  | 0.99 | 0.44 | 0.14 | 0.42 | 0.52 | 0.37 |  |  |  |  | 4 altered motifsf |
| rs2259816 |  | 0.99 | 0.44 | 0.14 | 0.42 | 0.52 | 0.37 |  | 5 tissues | LIV |  | 6 altered motifs |
| rs735396 |  | 0.99 | 0.44 | 0.14 | 0.42 | 0.52 | 0.37 |  | 6 tissues | THYM | POL2 | 4 altered motifs |

*Index SNPs. **AI – American Indians; AMR – Americans; AFR-African Americans; ASN- Asians; EUR-Europeans; IPSC-Induced pluripotent stem cells; LNG- Lung; LIV – Liver; BRST – Breast; BLD- Blood; PLCNT-Placenta; HRT-Heart, MUS-Muscle; KID-Kidney; GU-Gastrointestinal tract; PANC- Pancreas; ESDR- ; COMP-1- ; GR- ; HNF4- Hepatic nuclear factor 4; ER alpha - ; AIRE- RXRA -; MRSF; SP2-; PLZF; TCF12- Eggr-1; Ets- : Znf143 – Zinc finger protein 143
