# Supplementary figures and images for: Genetic analysis of hsCRP in American Indians: The Strong Heart Family Study
Source: PLoS One. 2019 Oct 17;14(10):e0223574. doi: 10.1371/journal.pone.0223574 (PMC6797125; doi:10.1371/journal.pone.0223574)

Chromosome 1  
Figure A

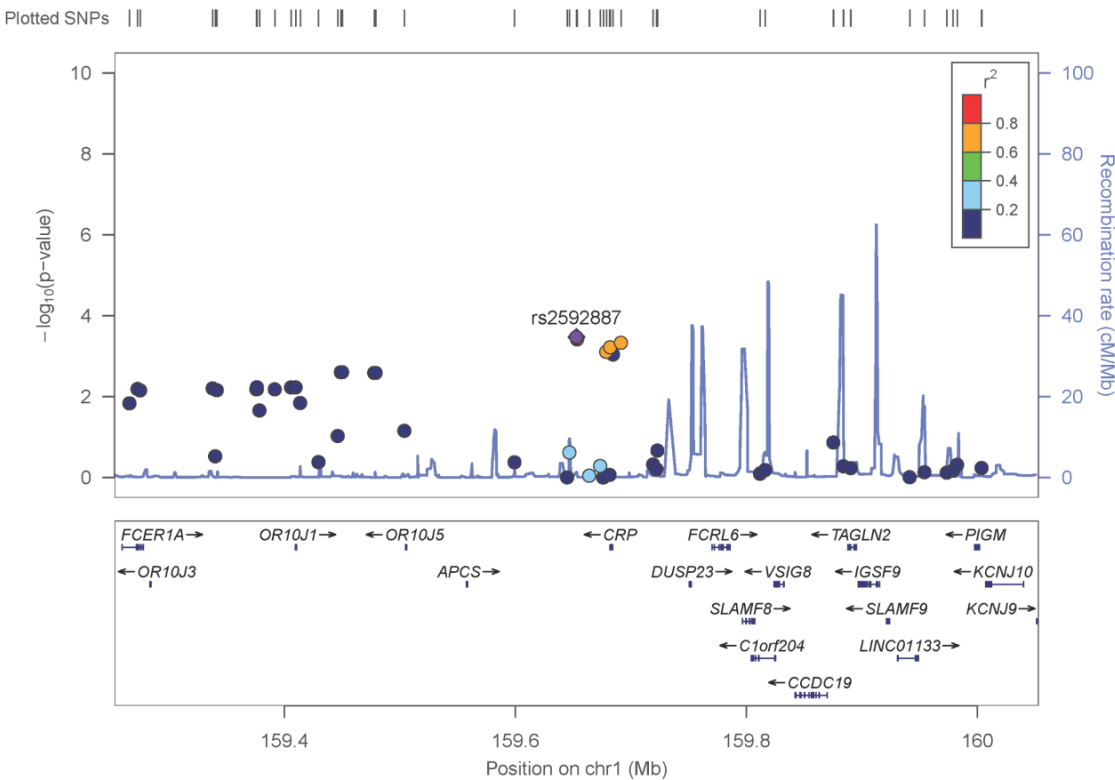

Chromosome 1  
Figure B

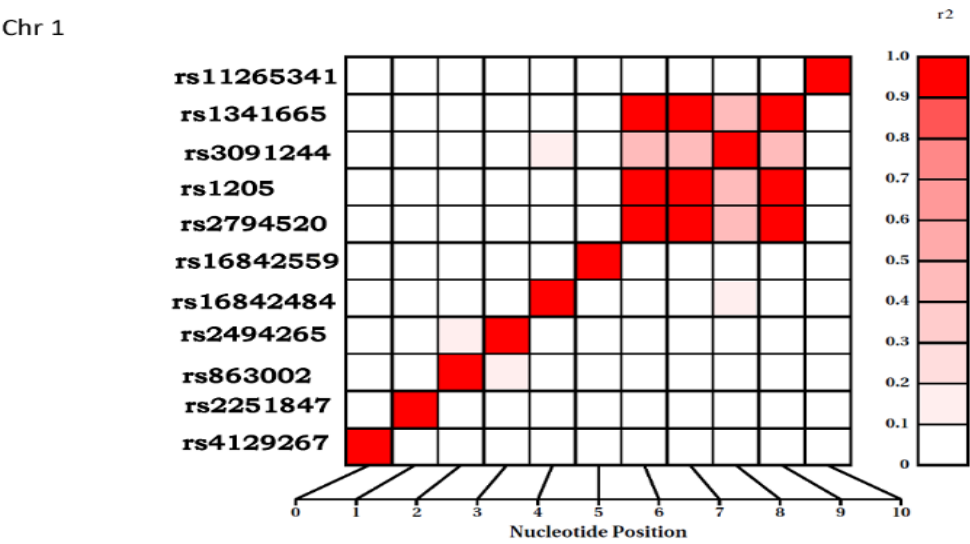

Supplement: S1 Fig — Locus zoom (A) and linkage disequilibrium plots (B) for chromosome 1. (PDF) [file pone.0223574.s005.pdf]

Chromosome 2  
Figure A

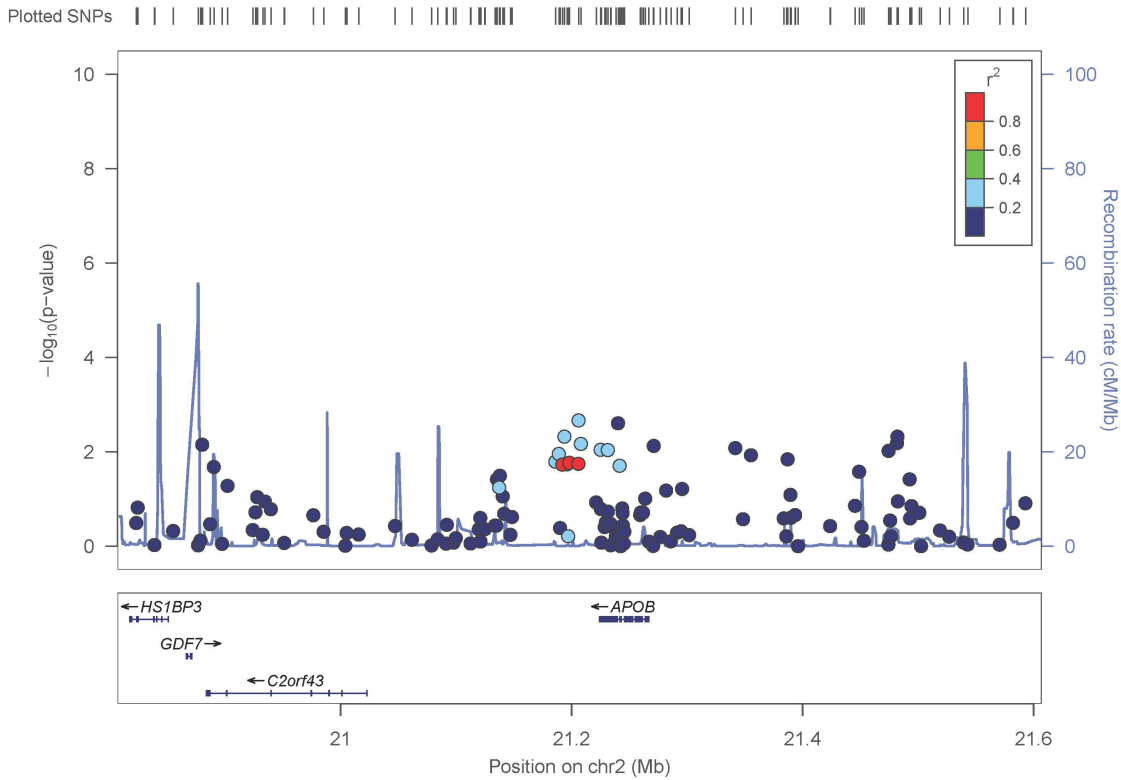

Chromosome 2  
Figure B

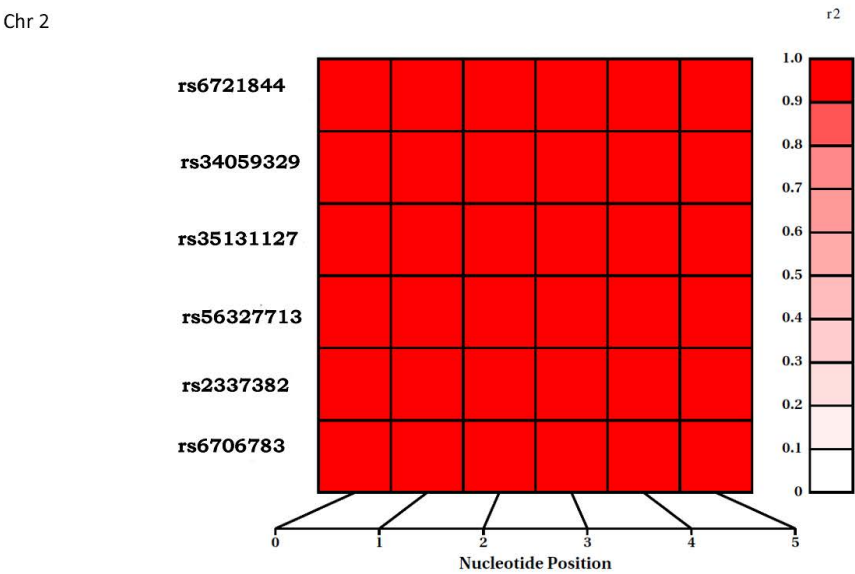

Supplement: S2 Fig — Locus zoom (A) and linkage disequilibrium plots (B) for chromosome 2. (PDF) [file pone.0223574.s006.pdf]

Chromosome 5  
Figure A

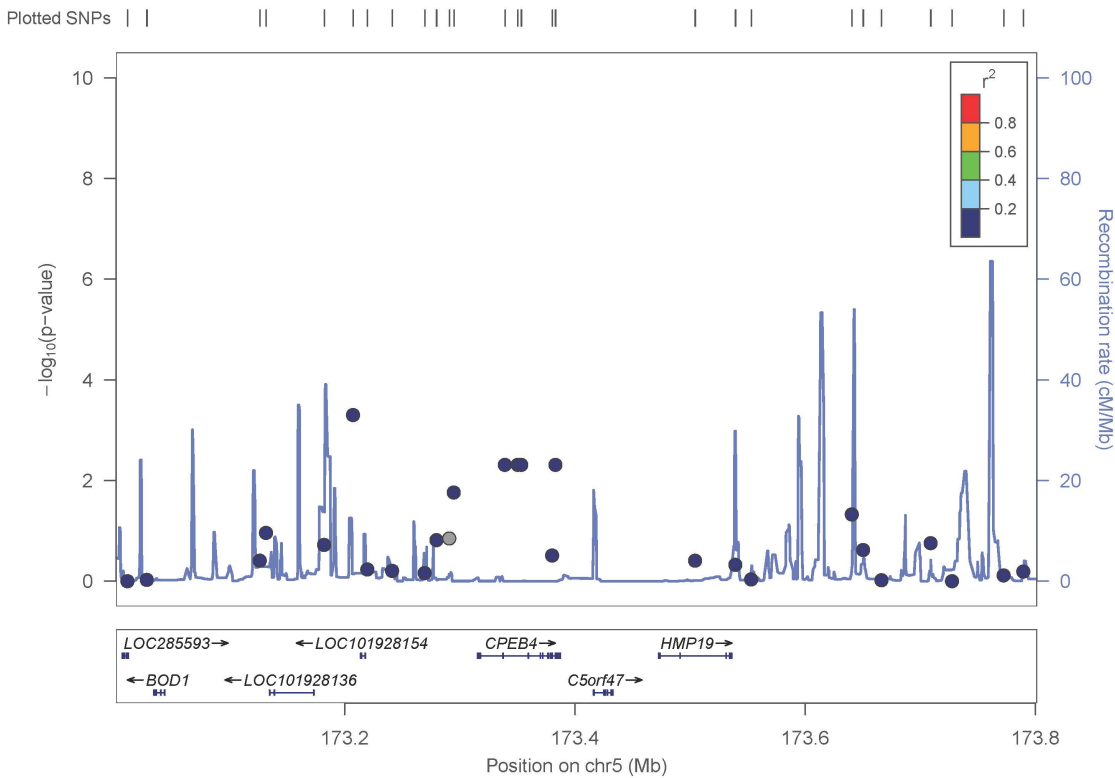

Chromosome 5  
Figure B

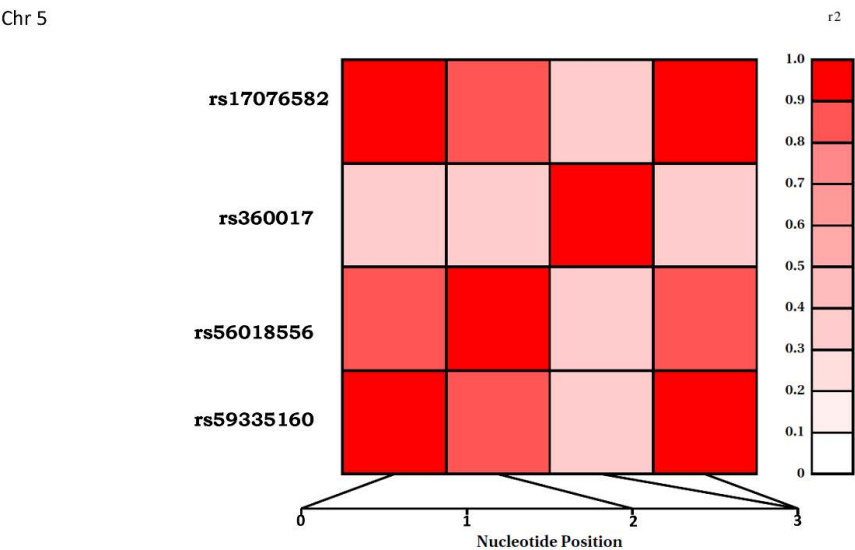

Supplement: S3 Fig — Locus zoom (A) and linkage disequilibrium plots (B) for chromosome 5. (PDF) [file pone.0223574.s007.pdf]

Chromosome 6a  
Figure A

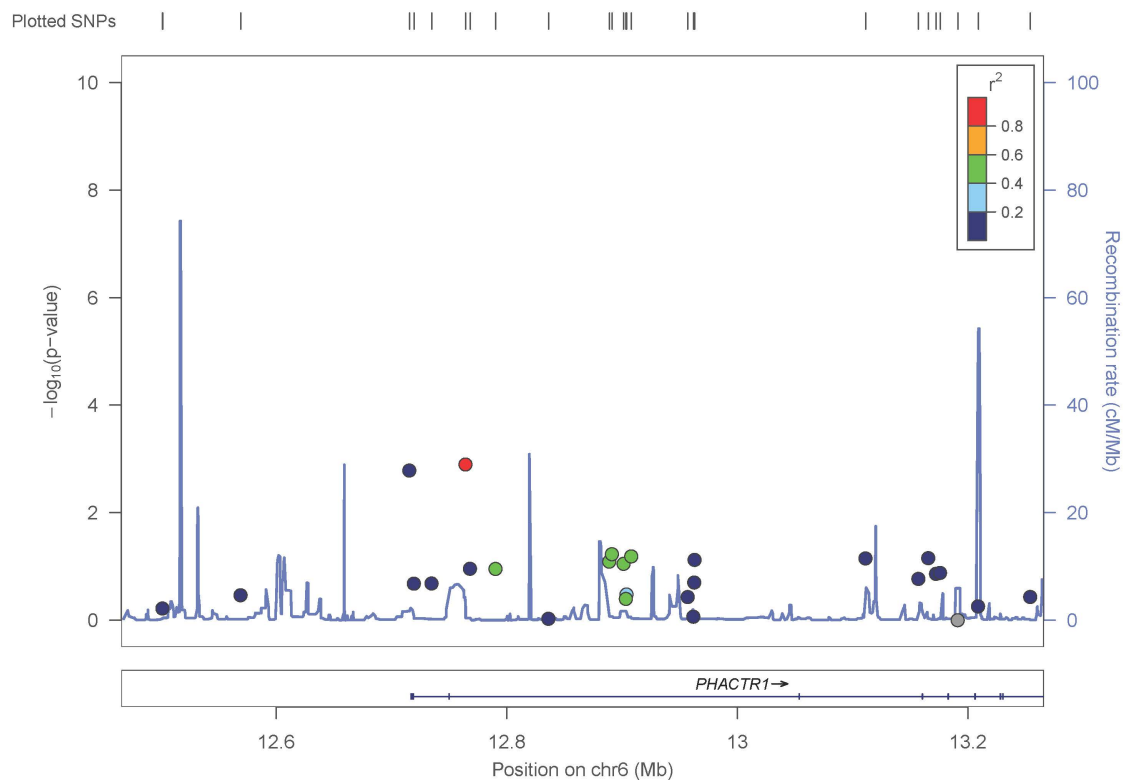

Chromosome 6a  
Figure B

Chr 6a

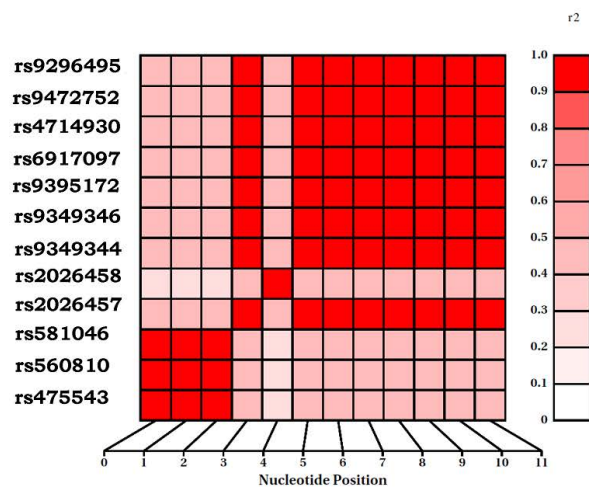

Supplement: S4 Fig — Locus zoom (A) and linkage disequilibrium plots (B) for chromosome 6a. (PDF) [file pone.0223574.s008.pdf]

Chromosome 6b  
Figure A

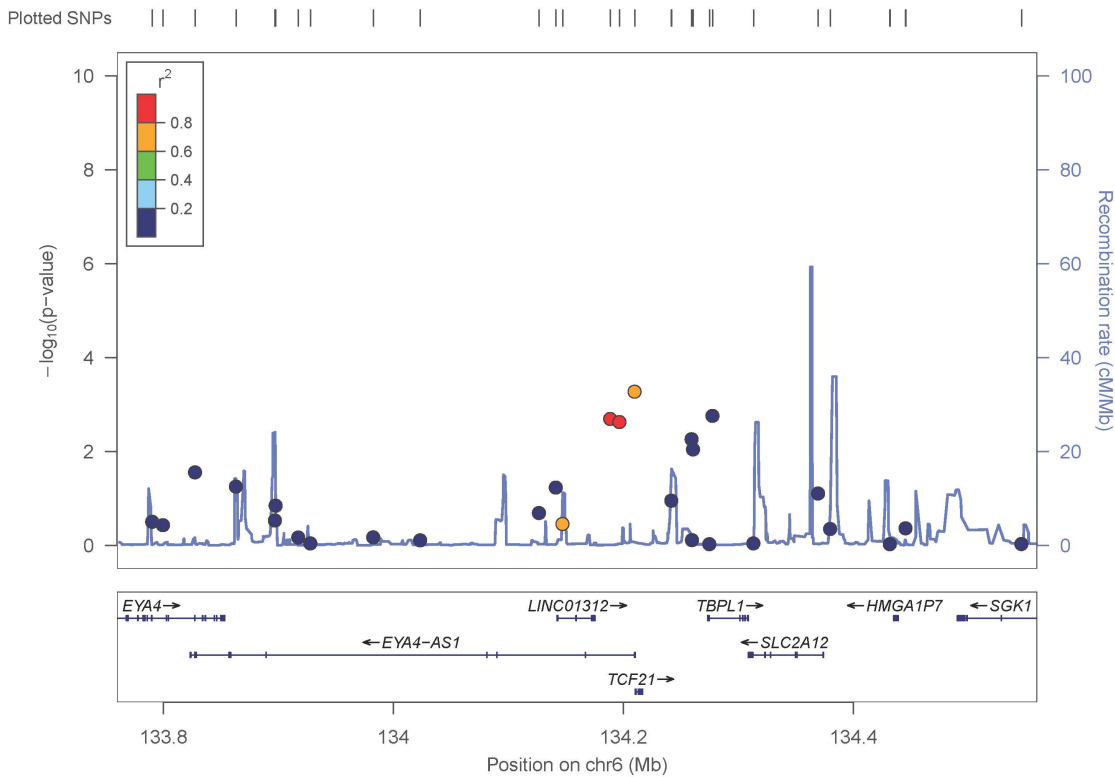

Chromosome 6b  
Figure B

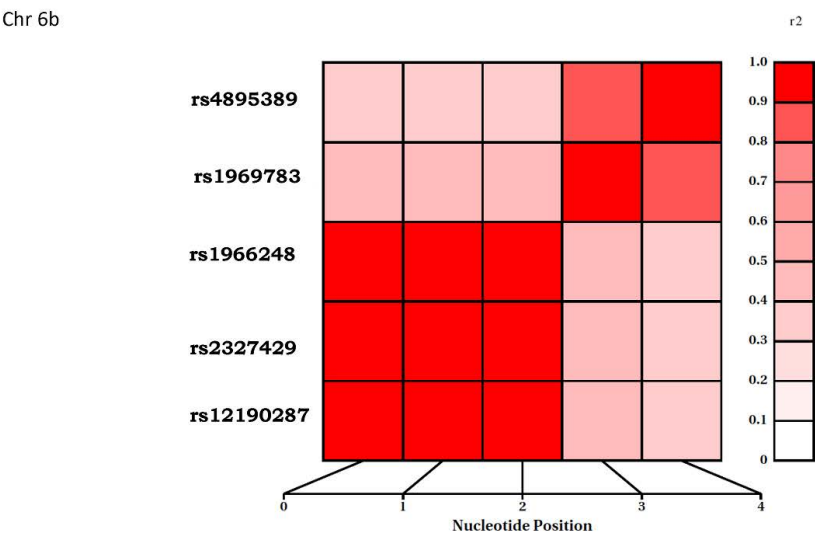

Supplement: S5 Fig — Locus zoom (A) and linkage disequilibrium plots (B) for chromosome 6b. (PDF) [file pone.0223574.s009.pdf]

Chromosome 9  
Figure A

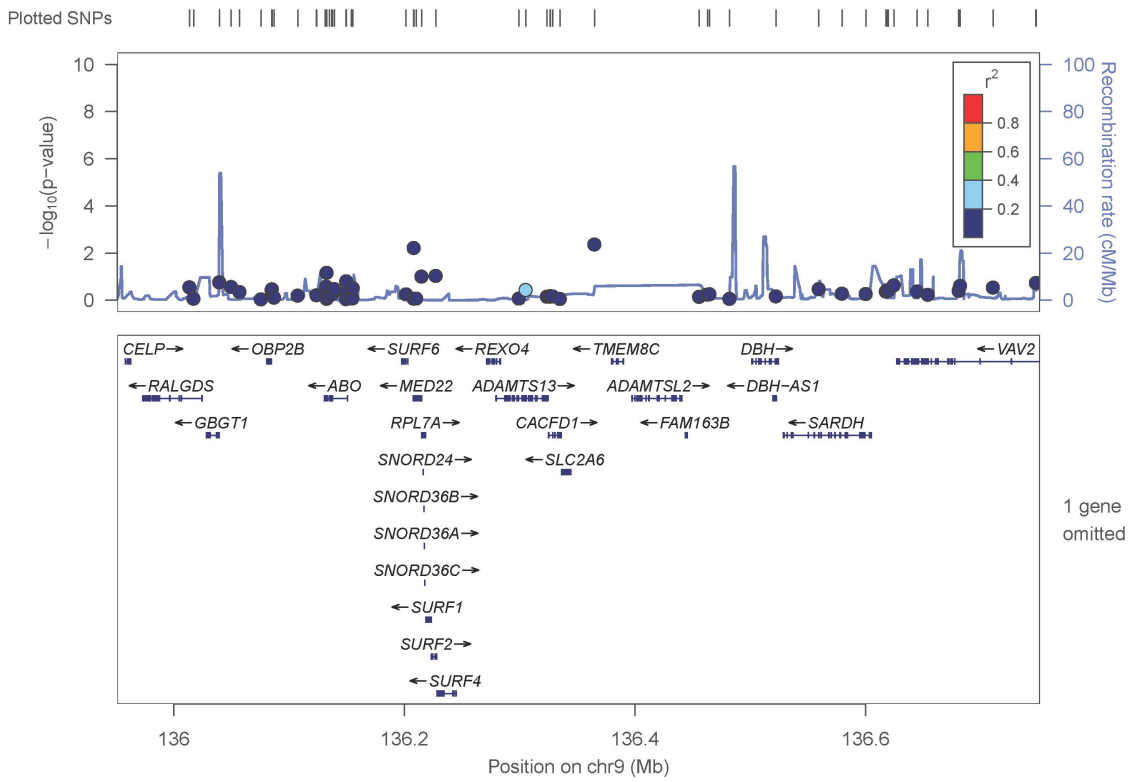

Chromosome 9  
Figure B

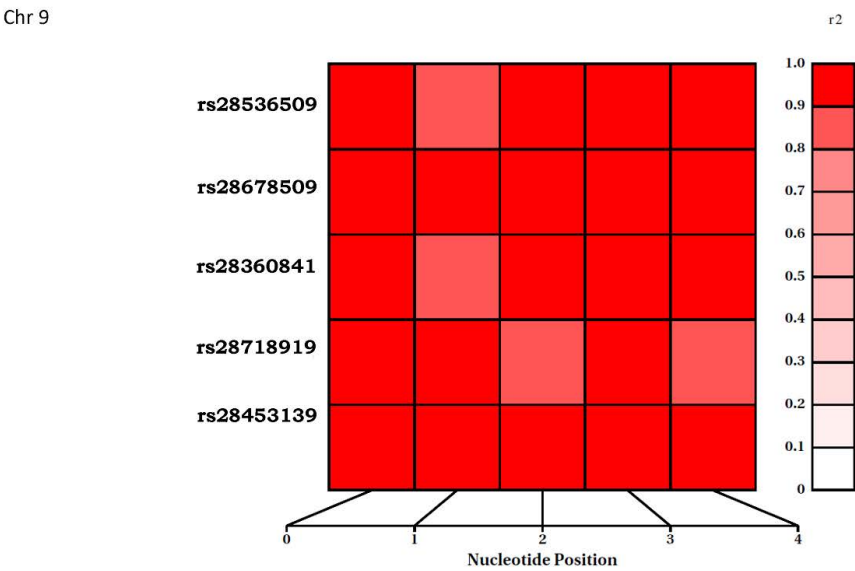

Supplement: S6 Fig — Locus zoom (A) and linkage disequilibrium plots (B) for chromosome 9. (PDF) [file pone.0223574.s010.pdf]

Chromosome 10  
Figure A

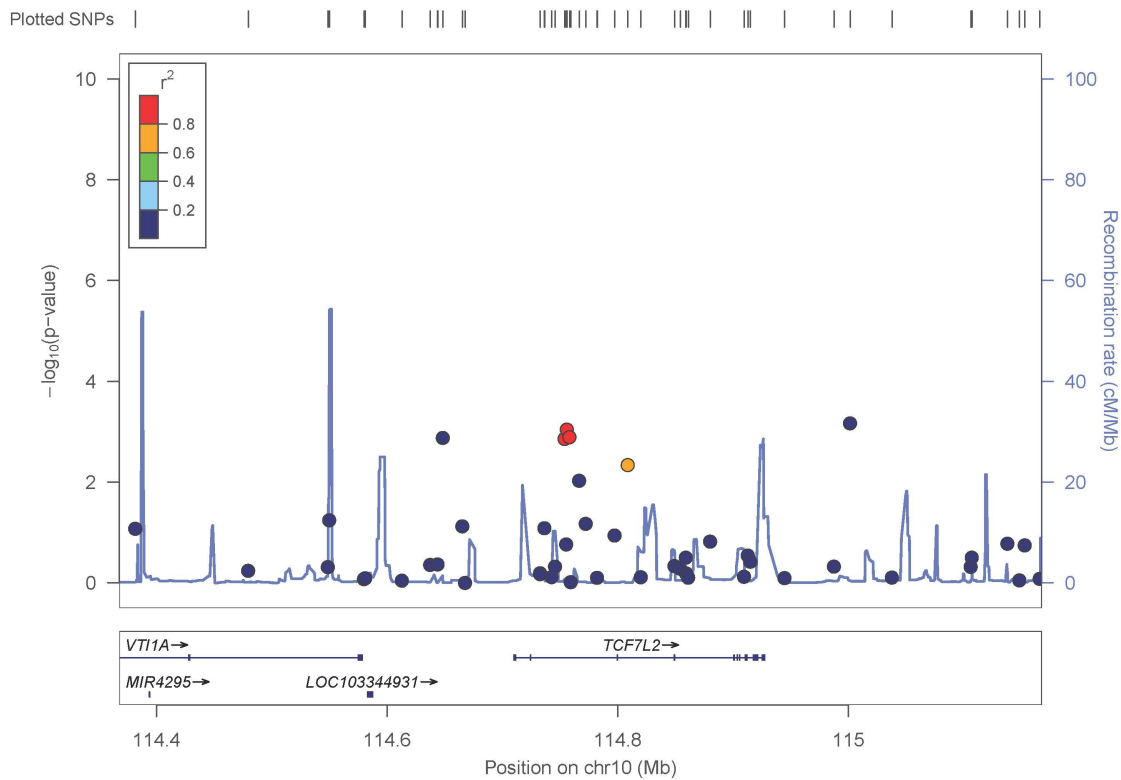

Chromosome 10  
Figure B

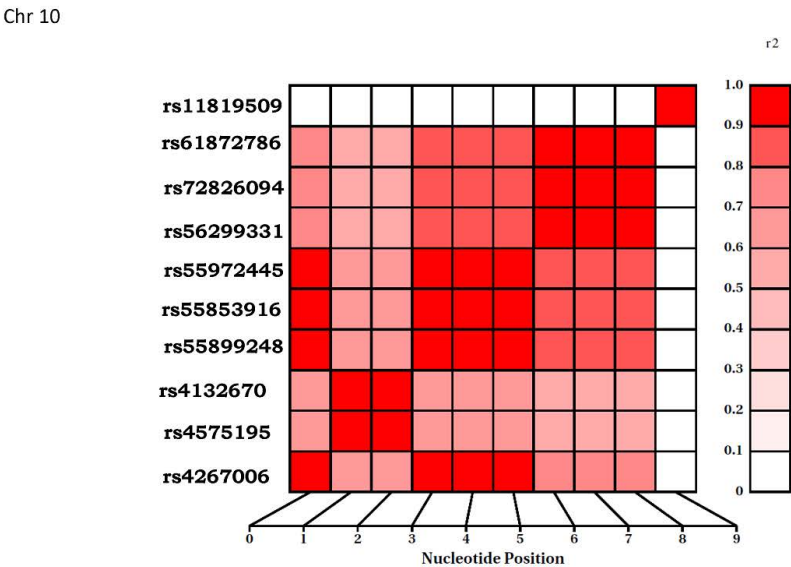

Supplement: S7 Fig — Locus zoom (A) and linkage disequilibrium plots (B) for chromosome 10. (PDF) [file pone.0223574.s011.pdf]

Chromosome 12  
Figure A

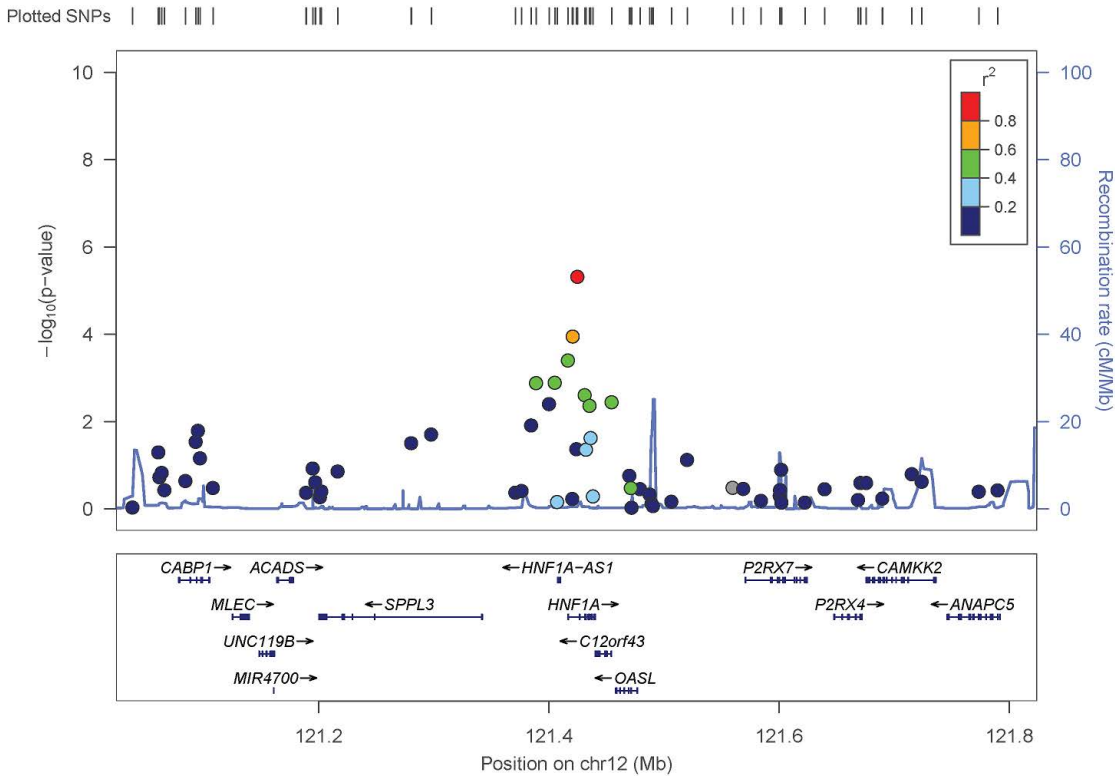

Chromosome 12  
Figure B

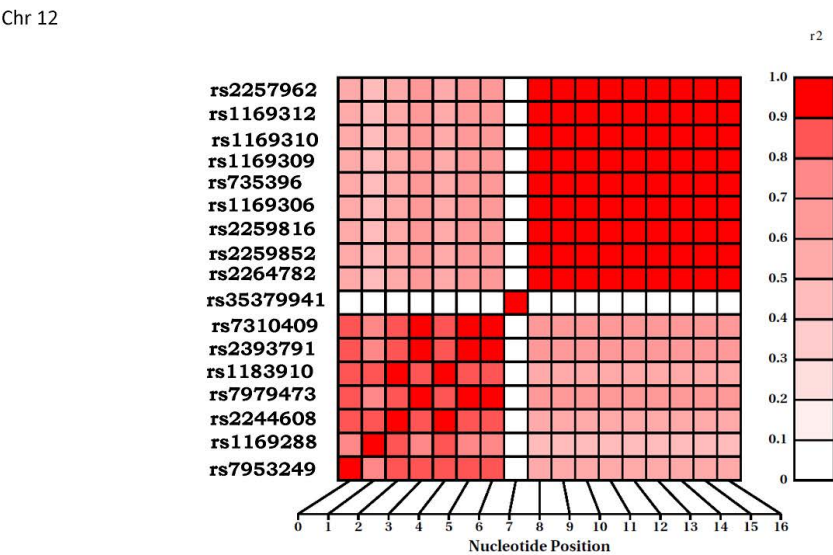

Supplement: S8 Fig — Locus zoom (A) and linkage disequilibrium plots (B) for chromosome 12. (PDF) [file pone.0223574.s012.pdf]

Chromosome 17  
Figure A

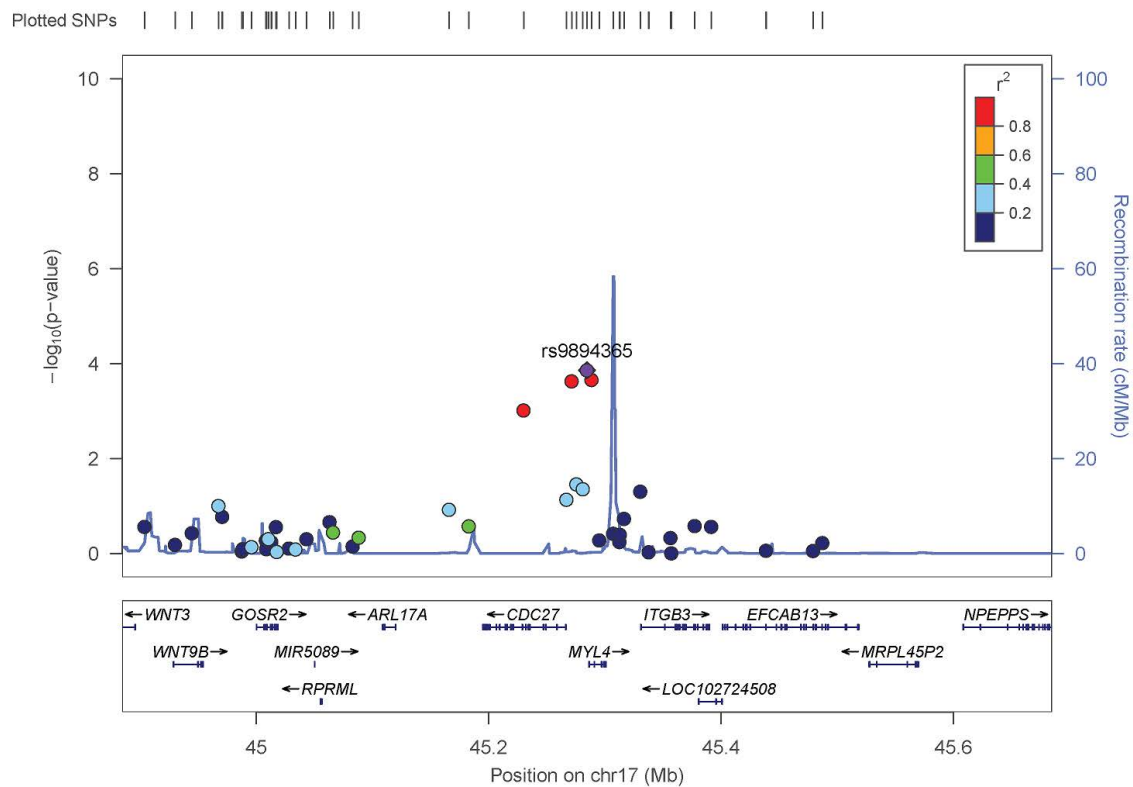

Chromosome 17  
Figure B

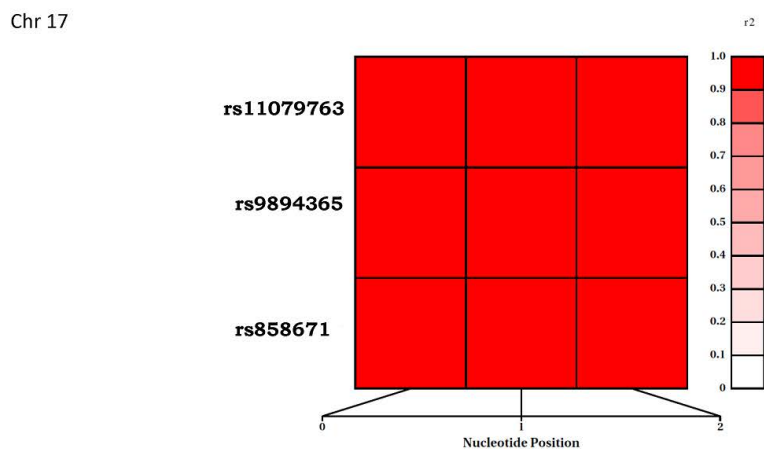

Supplement: S9 Fig — Locus zoom (A) and linkage disequilibrium plots (B) for chromosome 17. (PDF) [file pone.0223574.s013.pdf]

Chromosome 19  
Figure A

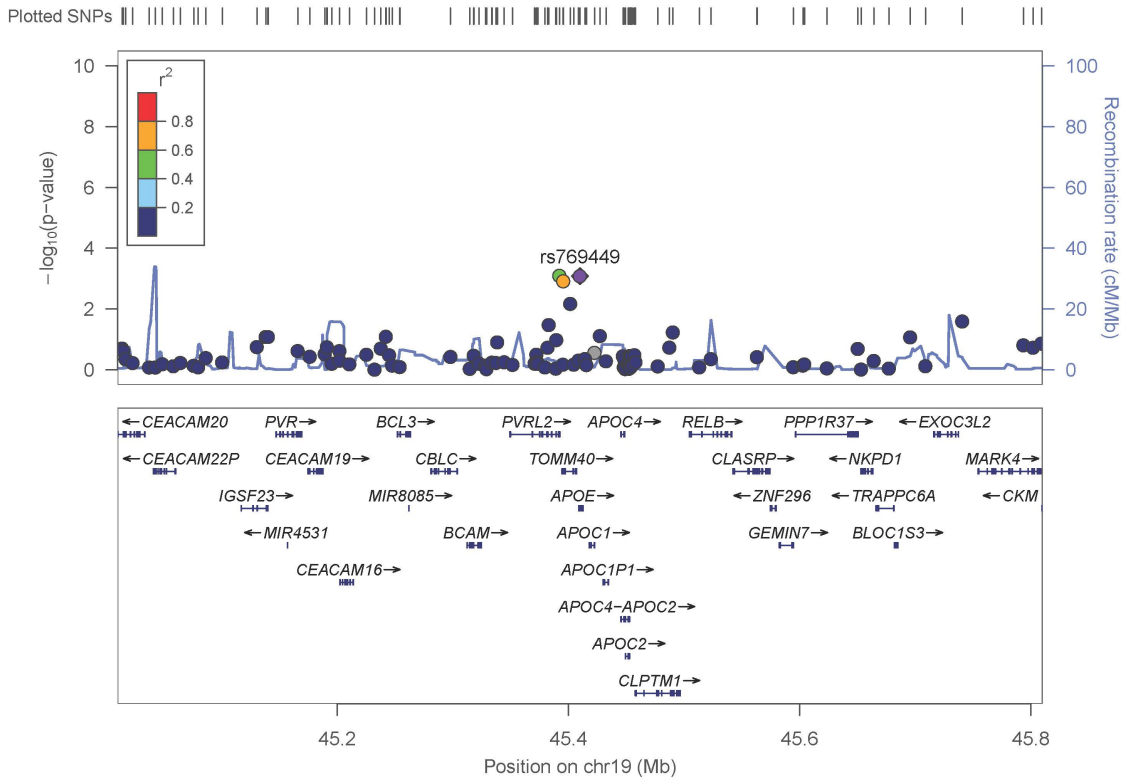

Chromosome 19  
Figure B

Chr 19

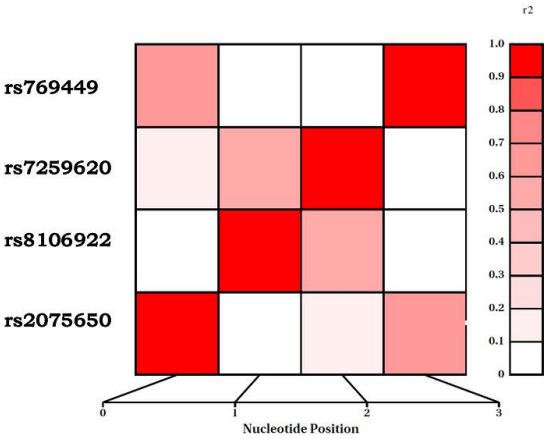

Supplement: S10 Fig — Locus zoom (A) and linkage disequilibrium plots (B) for chromosome 19. (PDF) [file pone.0223574.s014.pdf]
